# Does your species have memory? Analyzing capture–recapture data with memory models

**DOI:** 10.1002/ece3.1037

**Published:** 2014-04-30

**Authors:** Diana J Cole, Byron J T Morgan, Rachel S McCrea, Roger Pradel, Olivier Gimenez, Remi Choquet

**Affiliations:** 1 School of Mathematics, Statistics and Actuarial Science, University of KentCanterbury, UK; 2 Centre d'Écologie Fonctionnelle et Évolutive, Centre National de la Recherche ScientifiqueMontpellier, France

**Keywords:** Diagnostic goodness-of-fit tests, E-SURGE, identifiability, parameter redundancy, score tests, U-CARE

## Abstract

We examine memory models for multisite capture–recapture data. This is an important topic, as animals may exhibit behavior that is more complex than simple first-order Markov movement between sites, when it is necessary to devise and fit appropriate models to data. We consider the Arnason–Schwarz model for multisite capture–recapture data, which incorporates just first-order Markov movement, and also two alternative models that allow for memory, the Brownie model and the Pradel model. We use simulation to compare two alternative tests which may be undertaken to determine whether models for multisite capture–recapture data need to incorporate memory. Increasing the complexity of models runs the risk of introducing parameters that cannot be estimated, irrespective of how much data are collected, a feature which is known as parameter redundancy. Rouan et al. (JABES, 2009, pp 338–355) suggest a constraint that may be applied to overcome parameter redundancy when it is present in multisite memory models. For this case, we apply symbolic methods to derive a simpler constraint, which allows more parameters to be estimated, and give general results not limited to a particular configuration. We also consider the effect sparse data can have on parameter redundancy and recommend minimum sample sizes. Memory models for multisite capture–recapture data can be highly complex and difficult to fit to data. We emphasize the importance of a structured approach to modeling such data, by considering a priori which parameters can be estimated, which constraints are needed in order for estimation to take place, and how much data need to be collected. We also give guidance on the amount of data needed to use two alternative families of tests for whether models for multisite capture–recapture data need to incorporate memory.

## Introduction

Multisite capture–recapture studies record encounters with marked animals over several different sites. Generally, animals will return to the previously visited sites rather than randomly selecting a site. If this is the case, transitions will not be Markovian, so that the transition depends on where the animal was at the previous occasion, rather than just where the animal is at the present occasion. Such models are termed "memory models", see for example Brownie et al. (1993) and Rouan et al. (2009), and we also use this terminology here to represent multisite capture–recapture models where transitions are non-Markovian.

The multisite data set on the Canada Goose, *Branta canadensis*, from Hestbeck et al. (1991) has been used by a variety of authors to demonstrate the use of memory models (see for example Pradel et al. 2005). The Canada Goose data set is an example of a large multisite data set, having an average of around 1200 animals marked per year per site. In this article, we consider whether the memory model can be fitted to smaller data sets and whether diagnostic memory tests and score tests are able to detect memory when sample sizes are considerably smaller. The parameter redundancy of memory models has not previously been formally evaluated. However, Rouan et al. (2009) imposed a restriction to ensure that parameters could be estimated, but that was carried out in an arbitrary fashion and, as we show later in this article, a simpler, more effective constraint can be derived using formal procedures. Similarly, McCrea and Morgan (2011), who used score tests for model selection, while aware of parameter redundancy issues, did not provide checks using symbolic methods. The score tests that they used did not include the Pradel parameterization in the model set.

This study presents and extends various model diagnostics for memory models. Firstly, we consider which parameters can be estimated in these memory models. This involves examining whether or not models are parameter redundant (Cole et al. 2010). We can then examine, for a particular study, whether a species exhibits memory. This can be carried out through diagnostic goodness-of-fit tests (Pradel et al. 2005) or score tests (McCrea and Morgan 2011).

### Parameter redundancy

If two parameters are confounded, so that they only ever appear as a product in a model specification, then it will never be possible to estimate the two parameters individually; it will only be possible to estimate the product. A common example of this occurring in capture–recapture models is when survival and recapture probabilities are both time dependent. In that case, the survival and recapture probabilities for the last time-point are only ever seen as a product (see for example Cole et al. 2010). This problem is known as parameter redundancy. A parameter-redundant model will have at least one non-identifiable parameter. In practice, a model that is parameter redundant will cause problems with the estimation of parameters, because the likelihood surface will not posses a unique maximum and standard errors will not exist.

While parameter redundancy is obvious in some models, it is frequently not in others. There are several methods for investigating whether or not a model is parameter redundant, which include numerical methods (for example Viallefont et al. 1998), symbolic differentiation methods (for example Catchpole and Morgan 1997 and Cole et al. 2010), and hybrids of numeric and symbolic methods (Choquet and Cole 2012). Numeric methods alone can lead to incorrect conclusions regarding parameter redundancy (see for example Cole and Morgan 2010). Therefore, the use of symbolic or hybrids of numeric and symbolic methods is recommended as the most reliable methods for detecting parameter redundancy. More recently, the symbolic method has been extended to allow the use of symbolic methods in a wide range of complex models (Cole et al. 2010). Such theory has been used to investigate parameter redundancy in many ecological models, including ring-recovery models (Catchpole and Morgan 1997; Cole et al. 2010; Cole et al. 2012), capture–recapture models (Catchpole and Morgan 1997; Catchpole et al. 1998; Gimenez et al. 2004; Cole et al. 2010; Hubbard et al. 2014), capture–recapture-recovery models (Hubbard et al. 2014), and multistate models (Gimenez et al. 2003; Cole 2012).

### A diagnostic goodness-of-fit test for detecting memory

A diagnostic test, WBWA, was developed, by Pradel et al. (2003), to test for memory within multisite capture–recapture data. A series of contingency tables are constructed from the encounter information for each time *t*_*i*_, so that the number of individuals encountered in site *k* at time *t*_*i*−1_ and in site *r* at time *t*_*i*+1_ forms element (*k*, *r*) of the contingency table for occasion *t*_*i*_. Test WBWA (which stands for Where Before Where After) is then a standard test of homogeneity, as if no memory exists within the study (i.e., site at time *t*_*i*−1_ does not affect site at *t*_*i*+1_), then the test will be nonsignificant). We note that if large numbers of individuals do exhibit memory, they will tend to visit the same sites repeatedly, and then large observed numbers are expected on the diagonals of the contingency tables. This test is implemented in the computer package U-CARE (Choquet et al. 2009b), and we demonstrate how this is carried out in Supporting Information Data S1. Note that, this test is applied before model fitting, and therefore, it does not matter whether or not potential models are parameter redundant.

### Score tests

Score tests were first suggested by Rao (1948), and they provide a convenient alternative to likelihood-ratio tests for comparing nested models for a data set. Suppose model *M*_1_ is a simpler version of the more complex model *M*_2_. In order to compare the models using a likelihood-ratio test, it is necessary to fit both of the models to the data, in order to compare them using the values taken by the corresponding maximized log-likelihood values. By contrast, the same comparison made using score tests only requires the simpler of the two models to be fitted (*M*_1_), as the means of comparison involves derivatives of the log-likelihood which are zero at the maximum corresponding to model *M*_2_. If model *M*_2_ is inappropriate for the data, then it may be difficult to fit and that difficulty is typically avoided using score tests.

A range of statistical tests have been shown to be score tests, and, in particular, this is also true of certain diagnostic tests in capture–recapture (McCrea et al. 2014). The potential use of score tests in capture–recapture modeling in general was suggested by Morgan (1989), and examples were considered in detail by Catchpole and Morgan (1996). They advocated using score tests for model selection in a structured, step-up fashion, starting from simple models. The approach was shown to compare well with likelihood-ratio tests on a number of real ring-recovery data sets. The approach of using score tests in this way is particularly useful when the model set is large due to model complexity, as for instance arises with multisite models. This is demonstrated by McCrea and Morgan (2011) through the use of score tests for multisite mark–recapture model selection. They included a simulation study of performance, as well as an application to a real data set; they also included discussion of issues of multiple testing and also the use of step-down checks of the approach. See also McCrea et al. (2012), for multisite capture–recapture–recovery model selection, and Catchpole et al. (1999) who used score tests for variable selection in ring-recovery modeling.

Score test statistics require the computation of the expected information matrix, and Morgan et al. (2007) provide a simple illustration of the possible dangers of instead using an observed information matrix.

## Methods

### Models

We consider a general multisite capture–recapture study with *N* sites and *T* capture occasions and examine three different models, which are listed below, using the same notation as Rouan et al. (2009). The models have three different types of parameters: transition probabilities, initial state probabilities, and recapture probabilities. Transition probabilities incorporate both the probability of moving between sites and the probability of surviving from one occasion to the next.

- Model AS: This is the Arnason–Schwarz model (Arnason 1973; Schwarz et al. 1993), where transitions are Markovian; that is, the transition probabilities only depend on the current site and the site at the next occasion. The initial state probabilities are only dependent on the site the animal is in at that occasion. This model has no memory.
- Model B: This is the Brownie model (Brownie et al. 1993), which has non-Markovian transitions; that is, transition probabilities not only depend on the current and the site at the next occasion, but also the site the animal was in at the previous occasion. However, the initial state probabilities are only dependent on the site the animal is in at that occasion, resulting in separate Markovian transition probabilities for the first transition. This model allows for memory in transition probabilities for all but the first transition.
- Model P: This is the Pradel model (Pradel 2005), which also has non-Markovian transitions. This model allows for memory in all the transition probabilities. To allow the first transition to be non-Markovian, the initial state probabilities need to include where the animal would have been at the previous occasion as well as where the animal is at that occasion.

For all three models, the capture probabilities are only dependent on the site the animal is in at any occasion. The three types of parameters for each of the three models are summarized in Table1. We note that Hestbeck et al. (1991) were the first to investigate the idea of memory and their model is a special case of Model B.

**Table 1:** Parameters for the Arnason–Schwarz model (model AS), Brownie model (model B), and Pradel model (model P). Note that, transition probabilities include both movement between sites and survival from one year to the next. All parameters are probabilities, and *i*, *j*, and *k* refer to the site and range from 1 to *N*. The symbol † could also be used to replace *k* to indicate the animal is dead. The superscript *t* refers to the occasion. Note that, in models AS and B, 
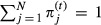
 and in model P, 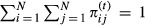. In model B, there are two options for transition probability: the 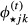 refers to the first capture when information is not known about the animal's previous location and the 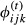 refers to subsequent ocassions

Model	Transition probability	Initial state probability	Recapture probability
AS	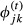 present at site *k* at *t* + 1 and at site *j* at *t*	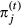 at site *j* when first captured at *t*	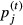 encountered alive at site *j* at *t*
B	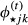 present at site *k* at *t* + 1 and at site *j* at *t* when first captured 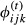 present at site *k* at *t* + 1 and at site *j* at *t* and *i* at *t*−1	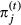 at site *j* when first captured at *t*	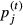 encountered alive at site *j* at *t*
P	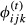 present at site *k* at *t* + 1 and at site *j* at *t* and *i* at *t*−1	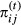 at site *j* when first captured at *t* and site *i* at *t*−1	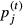 encountered alive at site *j* at *t*

We use standard notation for the recapture history of an animal, *h*, where 0 represents not encountered and *i* = 1,…,*N* represents encountered at site *i*. For example, the history


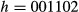


corresponds to a study over *T* = 6 occasions. The animal was first encountered at occasion *t* = 3 in site 1. It was then also encountered in site 1 at occasion *t* = 4. It was not encountered at occasion *t* = 5. Then, it was encountered at site 2 at occasion *t* = 6. We use *e* to denote the occasion the animal was first encountered; in this example, *e* = 3. To determine the probability of history *h*, we follow the matrix notation of Rouan et al. (2009). Here, the matrices represent the following *N* + 1 options: either an animal is in one of the *N* sites or the animal dies or permanently emigrates and moves to the "dead" state, denoted by †. There are three types of matrices: **Π**_*t*_, the initial state matrix; **Φ**_*t*_, the transition matrix; and **B**_*t*_, the event matrix. There is also an initial event matrix 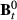 for the first encounter. The matrices for *N* = 2 sites are given in Table2, and matrices for a general *N* are given in Supporting Information Data S1. The probability of any encounter history *h* starting at time *e* can then be written as


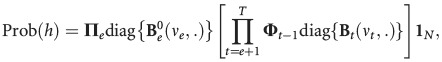


where *ν*_*t*_ is the event observed at time *t*, **B**_*t*_(*ν*_*t*_,.) is the row vector of **B** corresponding to event *ν*_*t*_, similarly 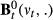 is the row vector of **B**^0^ corresponding to event *ν*_*t*_ and **1**_*N*_ is a column vector consisting of *N* ones. The term diag{**V**} refers to creating a diagonal matrix from row vector **V**. If there are *n* independent individual histories, the likelihood is then


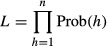


(Rouan et al. 2009).

**Table 2:** Matrices of probabilities used in the matrix notation for defining models AS, B, and P for *N*=2 site model. The symbol † refers to the dead state. In model AS, 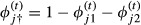. In model B 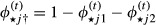. In models B and P, 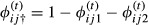. In addition, 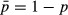 and Π^′^ represent the transpose of Π

Model	Initial state	Transition	Event
AS	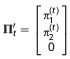	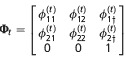	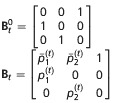
B	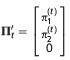	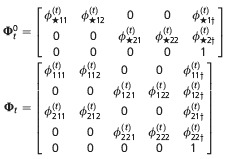	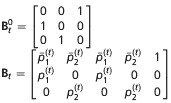
P	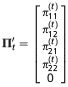	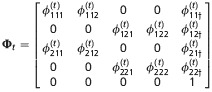	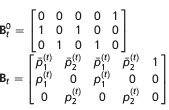

Memory models can be fitted using E-SURGE, which is a computer package for fitting multievent models (Choquet et al. 2009a). A guide to fitting memory models in E-SURGE is given in Supporting Information Data S1.

### Parameter redundancy

Parameter redundancy can be caused by the structure of the model, for example when two parameters are confounded. In such cases, regardless of the amount of data collected, all the parameters cannot be estimated. Parameter redundancy can also be due to there not being enough data (see for example Cole et al. 2012). We use two methods to investigate parameter redundancy: the symbolic method and the hybrid symbolic-numeric method. The former is used to obtain general results about parameter redundancy caused by the structure of the model itself. The hybrid symbolic-numeric method is used to investigate parameter redundancy due to the data.

#### Symbolic method

Memory models provide examples of models that are structurally too complex for use of the symbolic method developed in Catchpole and Morgan (1997). Instead, we need first to find a set of parameter combinations, which can be used to investigate parameter redundancy. Cole (2012) derived one such set of parameter combinations for multistate models using the methods described by Cole et al. (2010). In Supporting Information Data S1, a suitable set of parameter combinations is derived for multisite models. The parameter combinations for model B are given in equation (1) below; the combinations for model AS and model P are given in Supporting Information Data S1.


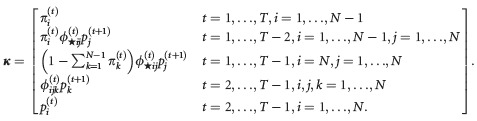
(1)

MAPLE code is given in Supporting Information Data S2 or S3 for automatically creating these sets of parameter combinations.

We demonstrate how to use equation (1) to investigate parameter redundancy by means of an example. Suppose that there are *N* = 2 sites, and none of the parameters are dependent on the capture occasion. For *T* = 3 occasions, the probability combinations are


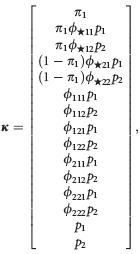


(where any repeated terms have been removed). We then form a derivative matrix, **D**, by differentiating each entry in ***κ*** in turn with respect to each of the parameters in the parameters vector


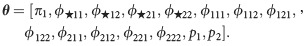


Note the order of differentiation is not important. This first terms of this derivative matrix are presented below


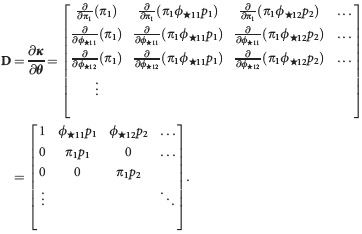


Next, we find the rank of the derivative matrix. The rank gives the number of parameters that can be estimated (Cole et al. 2010). In this case, the rank is 15 and there are 15 parameters in the model, so in this case, the model is not parameter redundant. We define the deficiency of the model as the number of parameters minus the rank, so in this case, the model has deficiency *d* = 0. Generally, a deficiency of *d* = 0 indicates that the model is not parameter redundant, whereas a deficiency of *d* > 0 indicates that a model is parameter redundant. For a parameter-redundant model, further symbolic algebra, involving solving a set of partial differential equations derived from the derivative matrix, will identify the parameters and combinations of parameters that can be estimated. These can form constraints that result in a model that is no longer parameter redundant. An example is provided in Supporting Information Data S1.

We can also use a result from Catchpole and Morgan (1997) to generalize these results to any number of years of data. Cole (2012) shows how this method of generalization can also be used to extend to any number of states, and we can similarly extend to number of sites. In the example above for *N* ≥ 2 sites and *T* ≥ *N* occasions, the rank of the derivative matrix is *N*^3^ + *N*^2^ + 2*N*−1, which is also the number of parameters, so the model will always have deficiency *d* = 0. The symbolic algebra involved can be carried out using a symbolic algebra package such as MAPLE (see for example Catchpole et al. 2002; Cole et al. 2010).

Note that any alternative parameterization of the models may be used. One such alternative, involving a reparameterization of the transition matrix to separate survival probability and movement probabilities between sites, is given in Supporting Information Data S1.

#### Hybrid symbolic-numeric method

The hybrid symbolic-numeric method (Choquet and Cole 2012) is similar to the symbolic method. However, rather than differentiating the elements of ***κ*** in equation (1), we differentiate the probabilities for each different encounter history present in a given data set. This allows the investigation of parameter redundancy for any particular data set. To find the rank of the resulting derivative matrix, it is evaluated numerically at a random point in the parameter space. Choquet and Cole (2012) recommend that this is carried out five times, at five different random points. The maximum of the five numerical ranks is taken as the number of estimable parameters. By considering all possible capture-histories, and not just those present in a data set, the hybrid symbolic-numeric method can also be used to investigate parameter redundancy caused by the model structure above.

The hybrid symbolic-numerical method can be implemented in E-SURGE or MAPLE. In Supporting Information Data S1, we provide a guide to how the hybrid method can be implemented in E-SURGE, and we also provide a MAPLE program that can be used to test whether a specific data set is parameter redundant or not using the hybrid symbolic-numeric method.

In this article, the symbolic method is used to find general results about a model. For example, the general result for the example above is that the deficiency of model B is always *d* = 0. The general result will be the smallest possible deficiency, as it is based on the assumption that all possible histories are observed. Of course, in many real data sets, all the possible histories are not observed, and we shall use the hybrid symbolic-numeric method to investigate the size of a data set that is required for the general parameter redundancy result to still hold.

We now outline an heuristic approach to indicate the likely sample sizes needed in order for the symbolic results to hold. For specific parameter values, we can find the probability of any history *h* occurring, *P*(*h*). If *m* animals are marked each year, at each of the *N* sites, then we expect to see *E*(*h*) = *m* × *N* × *P*(*h*) animals with history *h*. For example, consider model B with *T* = 3 years and *N* = 2 sites and parameter values *π*_1_ = 0.1, *p*_*i*_ = 0.2, *ϕ*_⋆*ij*_ = 0.3, and *ϕ*_*ijk*_ = 0.3 for *i*,*j*,*k*=1,2. The probability of the history *h* = 001 is *P*(001) = *π*_1_ = 0.1, and if there were *m* = 10 animals marked per year per site, we would expect to see the history *h* = 001 twice as *E*(001) = 10 × 2 × 0.1 = 2. Whereas the probability of the history *h* = 101 is *P*(101) = *π*_1_ϕ_*11_(1−*p*_1_)*ϕ*_111_*p*_1_+*π*_1_ϕ_*12_(1−*p*_2_)*ϕ*_121_*p*_1_ = 0.00288, and if there were *m* = 10 animals marked per year per site, we would not expect to see the history *h* = 101 as *E*(101) = 10 × 2 × 0.00288 = 0.0576. Using these expected values, we can create an "expected data set". To explore parameter redundancy caused by the data, we only need to consider whether a history is present or absent from a data set. When *E*(*h*) is greater than or equal to 1, we suppose the history is present in our "expected data set". When *E*(*h*) is less than 1, we suppose the history is not in our expected data set. In this example, the "expected data set" is *h*_1_ = 100, *h*_2_ = 200, *h*_3_ = 010, *h*_4_ = 020, *h*_5_ = 001, *h*_6_ = 021, *h*_7_ = 002, and *h*_8_ = 022. In this case, the deficiency is *d* = 9, whereas the general result when all histories are present is *d* = 0.

It is then possible for specific parameter values to determine the smallest value of *m* required for the "expected data set" to result in the same deficiency as the general result. To provide recommendations on the amount of data needed to obtain the smallest deficiency possible, we create multiple "expected data sets" for different numbers *N* and *T* and for different parameter values. For each "expected data set", we determine the smallest value of *m* for the general result to apply.

### Investigating tests for memory

In this article, we consider two different tests for deciding if there is memory or not: the WBWA test and score tests. The WBWA test requires no model fitting. The score tests only require fitting the model without memory.

We investigate how large a data set is required for the WBWA and score tests to detect memory in a data set using simulation. There are two alternatives for the score tests. In both cases, the null hypothesis is that the data are adequately modeled by model AS. The alternative hypothesis can either be model B or model P.

We consider two instances of a two-site model: when there is memory (*M*) and when there is not memory (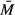). The simulated data sets with memory have transition matrix


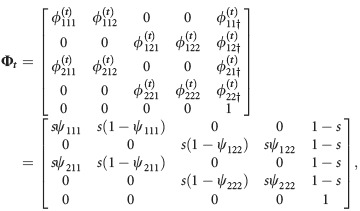
(2)

where *s* is the probability of surviving from one occasion to the next and *ψ*_*ijj*_ is the probability an animal stays at the same site *j* given that it was at site *i* on the previous occasion. For the simulation, we set the parameters to *π*_11_ = *π*_12_ = *π*_21_ = 0.25, *s* = 0.9, *ψ*_111_ = 0.7, *ψ*_211_ = 0.3, *ψ*_222_ = 0.6, *ψ*_122_ = 0.4, *p*_1_ = 0.5 and *p*_2_ = 0.3, so that there is a higher probability of staying at a site if the animal was at that site at the previous occasion compared with the alternative. For the simulated data sets without memory, the transition matrix is


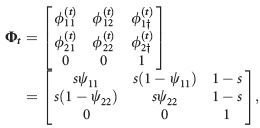
(3)

and the parameter values used are *π*_1_ = 0.5, *s* = 0.9, *ψ*_11_ = 0.8, *ψ*_22_ = 0.6, *p*_1_ = 0.5 and *p*_2_ = 0.3. We suppose that there are *T* = 10 years of data and that *m* = 25, 50, 75, 100, 125, 150 new animals are marked each year at each site, so that total sample sizes are 500, 1000, 1500, 2000, 2500, and 3000, respectively. For each value of *m*, 100 data sets are simulated. In Supporting Information Data S1, we describe a similar simulation study for *N* = 3 sites. Note that, the parameters here have been chosen for illustration, and different results would be obtained with different parameter values.

## Results

### Parameter redundancy

Using the symbolic method, we can obtain general results about parameter redundancy for particular models. Illustrative general results are given in Table3. We consider allowing each of the transition, initial state and recapture probabilities to be constant over each occasion or time dependent.

**Table 3:** (a) Deficiency of various AS, B and P models. A deficiency of zero means the model is not parameter redundant. A deficiency greater than 0 mean the model is parameter redundant. C, constant parameters, T, time-dependent parameters. (b) The range of sample sizes per site per year needed to achieve the general parameter redundancy results in the first half of the table. *N* is the number of sites

*π*	*ϕ*	*p*	Model AS	Model B	Model P
(a) Deficiency					
C	C	C	0	0	0
C	C	T	0	0	0
C	T	C	0	0	*N*^3^−*N*^2^
C	T	T	*N*	*N*	*N*^3^−*N*^2^ + *N*
T	C	C	0	0	(*N*−1)^2^
T	C	T	0	0	(*N*−1)^2^
T	T	C	0	0	*N*^3^ + *N*^2^−3*N* + 1
T	T	T	*N*	*N*	*N*^3^ + *N*^2^−2*N* + 1
(b) Recommended sample size					
C	C	C	(5, 15)	(10, 60)	(5, 30)
C	C	T	(5, 25)	(10, 70)	(10, 30)
C	T	C	(10, 165)	(35, 500)	(20, 150)
C	T	T	(20, 165)	(45, 500)	(30, 250)
T	C	C	(5, 30)	(10, 75)	(10, 50)
T	C	T	(10, 35)	(15, 75)	(15, 150)
T	T	C	(20, 330)	(50, 500)	(30, 305)
T	T	T	(25, 330)	(55, 610)	(45, 305)

We note that the AS and B models are only parameter redundant when both *ϕ* and *p* are time dependent. It is then not possible to estimate individually probabilities at the last time-points but it is possible to estimate their product. Thus, in model AS, it is not possible to estimate 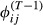 and 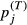, but it is possible to estimate 
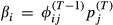
 for *i* = 1,…,*N*. For model B, it is not possible to estimate 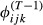 and 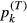, but it is possible to estimate 
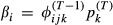
 for *i*,*j* = 1,…,*N*. Applying this constraint for all parameter-redundant AS and B models of Table3 results in models that are no longer parameter redundant.

The confounding for model P is more complex. More of the models that are considered in Table3 are parameter redundant. As well as the last time-point probabilities, *ϕ*^(*T*−1)^ and *p*^(*T*)^ being confounded, there is also confounding in the probabilities of the first time-points for *ϕ*^(1)^ and *π*^(1)^ and last time-point for *π*^(*T*)^. To ensure all parameters can be estimated, Rouan et al. (2009) suggest constraints on all the *π*^(*t*)^ parameters as well as *ϕ*^(1)^ and *ϕ*^(*T*)^. They showed that these constraints were effective for *N* = 2 and 3 and *T* = 4, 5, and 6. Using the symbolic methods described in Section 2.2, it can be shown that their constraints always result in a model with deficiency zero. By solving an appropriate set of partial differential equations, we find a simpler variation in the constraint that does not constrain all the *π*^(*t*)^ parameters.


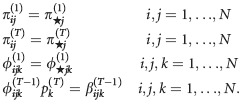


These constraints present a substantial gain, compared with the constraints of Rouan et al. (2009), as they allow (*N*^2^−1)(*T*−2) more of the original parameters to be estimated. The results are also completely general.

Table3 also provides a guide to the range of numbers of animals that need to be marked at each site each year for the general parameter redundant results to remain unchanged. If the average number of animals marked per year per site is greater than the upper limit of the range, then the deficiency for most examples will remain unchanged compared with the general result. Then, there should be no problems with fitting the model, as long as for the parameter redundant models, the above constraints are used. However, this is based on the assumption that the data follow the underlying memory model; if this is not the case, then an atypical data set could still be parameter redundant behold the upper limit. If the average number of animals marked per year per site is within the range, then the deficiency may change depending on the underlying parameter values. It is recommended in this case that the MAPLE code in Supporting Information Data S4 or S5 be used to check the deficiency for the specific data set used. If the average number of animals marked per year per site is less than the range, then the data set is very likely to have a lower deficiency compared with the general result.

For the simplest case with no time-dependent parameters, a very small number of an animals can be marked per site per year and it is still possible to fit a memory model. For more complex models, larger sample sizes are needed; however, we do not need as large a sample size as the Canada Goose data set. Model AS needs the smallest sample sizes for deficiency to remain unchanged, then Model P, with model B needing the largest sample sizes. This corresponds to the number of parameters in the respective models, models with more parameters need larger sample sizes, as we might expect. From the results in Table3(b), we deduce that for smaller data sets, it may be preferable to use model P with the above constraints rather than model B.

### Tests for memory

Simulations have been run in order to evaluate how well the WBWA and score tests perform. In Table4, we present the percentage of simulations that gave the wrong result for a 5% significance level. For simulated data sets with memory, we present the percentage of simulations whose *P*-value is greater than or equal to 0.05, whereas for simulated data set without memory, we present the percentage of simulations whose *P*-value is less than 0.05. In both cases, percentages of around 5% or less indicate that the test is performing well. Other statistics from the simulation studies are given in Supporting Information Data S1.

**Table 4:** The percentage of simulations that gave the wrong conclusion under a 5% significance, split by whether the simulation had memory, (a) or did not have memory (b). In the simulation, *m* is the number of animals marked per year per site and *N* is the number of sites. WBWA refers to the WBWA test. Score B refers a score test comparing model AS with model B. Score P refers to a score test comparing model AS with model P

	*N* = 2			*N* = 3		
		
m	WBWA, %	Score B, %	Score P, %	WBWA, %	Score B, %	Score P, %
(a) Simulation with memory						
25	84	8	3	96	25	11
50	60	0	0	43	5	5
75	31	0	0	26	2	0
100	14	0	0	14	4	2
125	7	0	0	3	4	3
150	6	0	0	1	3	2
(b) Simulation without memory						
25	0	10	7	1	23	14
50	1	4	5	5	4	6
75	2	9	6	3	4	3
100	3	5	4	6	1	1
125	2	4	6	5	7	1
150	7	8	9	8	6	3

It is clear that the WBWA test is not picking up memory correctly for *m* = 25, 50, and 75. The test starts to preform better when *m* = 100, but is only doing as well as it would be expected when *m* = 125 and 150. The score test however preforms as expected for sample sizes of *m* ≥ 50.

The poor power of the WBWA test for small sample sizes is mostly likely due to the conditioning of data required for the test to be performed: only individuals that are encountered on three consecutive occasions contribute to the test statistic and therefore not only is the power of the test sensitive to small sample sizes, but also parameter values such as capture probabilities.

## Discussion

Before fitting a memory model, it is important to consider exactly what can be estimated in a model. If a parameter-redundant model is fitted to the data, then 1) either the model fitting will fail, the standard errors will not exist or be very large or 2) the model fitting does not fail but wrong parameter estimates and standard errors are returned. It is therefore recommended that the parameter redundancy of a model is examined before model fitting is considered. Here, we have provided the tools for examining parameter redundancy in models AS, B, and P. In parameter-redundant models, it is possible to discover exactly what can be estimated and find suitable constraints that result in a model that is no longer parameter redundant. In Section 3.1, we have also shown how to obtain general results for any number of occasions and sites.

We have also considered how many animals need to be marked per year per site for results to hold. To ensure whether the memory model, with no time-dependent parameter, is not parameter redundant, then we would recommend marking at least 30 animals per year per site if model P is used, or at least 60 animals per year per site, if model B is used; however, for the equivalent model without memory, model AS, we would recommend marking at least 15 animals per site per year. These numbers increase when any of the parameters are dependent on sampling occasion. For smaller sample sizes, model P with the constraints given in Section 3.1 would be better in terms of parameter redundancy than model B. What is clear from this study is that we do not need data sets as large as the Canada Goose data set to use memory models.

In terms of general parameter redundancy, model B has more models with deficiency 0, so would be the preferred model to fit. However, we have given constraints that allow model P to still be used. Model P does better than model B in terms of the sample size needed to achieve the general results. Therefore, model P with constraints may be preferable to model B for smaller sample sizes.

To use the WBWA test, it is recommended that a sample size of at least 100 animals marked per site per year is used. Whereas to use a score test, 50 animals per year per site is sufficient for the score test to identify memory. The score test is better than the WBWA test at identifying memory in smaller data sets. However, the diagnostic test is easy to apply as it is available in U-CARE. Bespoke MATLAB code was written for the score test. The two approaches also employ a different strategy for subsequent model selection, with diagnostic tests potentially suggesting a range of possible model extensions relative to the AS model, all of which then need consideration in a second stage of model fitting and comparison. By contrast, score tests which start with a test of memory can then be developed through a succession of step-up stages, involving model elaboration, each time only fitting the most significant model (see for example McCrea and Morgan 2011).

We note finally that our focus in this article has been on exploring tests for memory and considering the parameter redundancy of appropriate models. We have not presented a complete model selection process, which would involve a combination of the different tools in the article.

## Supporting Information

**Data S1.** Electronic appendix.

**Data S2.** Maple code to for automatically creating sets of estimable parameter combinations for models AS, B and P.

**Data S3.** Pdf version of Maple code to for automatically creating sets of estimable parameter combinations for models AS, B and P.

**Data S4.** Maple code examples.

**Data S5.** Pdf version of Maple code examples.

**Data S6.** Example simulated data set with memory.

**Data S7.** Example simulated data set without memory.
