# Supplementary material for: Does your species have memory? Analyzing capture–recapture data with memory models
Source: Ecol Evol. 2014 Apr 30;4(11):2124–33. doi: 10.1002/ece3.1037 (PMC4201427; doi:10.1002/ece3.1037)
Supplement: Supplementary file 1 — Data S1. Electronic appendix. [file ece30004-2124-sd1.pdf]

Supplementary Material for the Paper: Does Your Species Have Memory? Analysing Capture-Recapture Data with Memory Models.

Cole, D. J.
School of Mathematics, Statistics and Actuarial Science,
University of Kent, Canterbury, England,
Morgan, B. J. T.
School of Mathematics, Statistics and Actuarial Science,
University of Kent, Canterbury, England,
McCrea, R. S.
School of Mathematics, Statistics and Actuarial Science,
University of Kent, Canterbury, England,
Pradel, R.
Centre d'Écologie Fonctionnelle et Évolutive,
Centre National de la Recherche Scientifique, Montpellier, France.
and
Gimenez, O.
Centre d'Écologie Fonctionnelle et Évolutive,
Centre National de la Recherche Scientifique, Montpellier, France,
Choquet, R.
Centre d'Écologie Fonctionnelle et Évolutive,
Centre National de la Recherche Scientifique, Montpellier, France,

1 Introduction

In this supplementary material we provide further information on the memory model, parameter redundancy, diagnostic goodness of fit tests and score tests.

2 Memory Model

As stated in the main paper the probability of any encounter history h starting at time e can then be written as

$$\text{Prob}(h) = \mathbf{\Pi}_e \text{diag}\left\{\mathbf{B}_e^0(\nu_e, .)\right\} \left[\prod_{t=e+1}^{T} \mathbf{\Phi}_{t-1} \text{diag}\left\{\mathbf{B}_t(\nu_t, .)\right\}\right] \mathbf{1}_N,$$

where ν_t is the event observed at time t, $\mathbf{B}(\nu_t, .)$ is the row vector of $\mathbf{B}$ corresponding to event ν_t, and $\mathbf{1}_N$ is a column vector consisting of N ones. In Table 2 of the main paper we give the matrices $\mathbf{\Pi}_t$, $\mathbf{\Phi}_t$ and $\mathbf{B}$ for $N = 2$ states. Table 1 gives these matrices for a general N. (The matrices for $N = 3$ are given in Rouan et al, 2009.)

3 Parameter Redundancy

To investigate parameter redundancy in structurally complex models, such as multi-site capture-recapture models, Cole et al (2010) generalise the symbolic method first used for ecological models in Catchpole and Morgan (1997). In the main paper we demonstrated how to use a particular vector of parameter combinations to determine whether or not a model is parameter redundant. This method involves differentiating the vector of parameter combinations with respect to the parameters to form a derivative matrix, and then calculating the rank of that derivative matrix. This vector of parameter combinations is called an exhaustive summary in Cole et al (2010).

An exhaustive summary is as a vector of parameter combinations that uniquely defines a model; (a more formal definition is given in Cole et al, 2010). Essentially an exhaustive summary is a vector of parameter combinations that can be used to infer results about parameter redundancy. For any model there will be many different options for exhaustive summaries. An exhaustive summary that can be used in capture-recapture models is the set of probabilities of life histories. This could be the probabilities of every possible life history if interest lies in the parameter redundancy of the model, or it could be just the probabilities of the life histories in a particular data set if interest lies in parameter redundancy caused by the data and the model together. Another exhaustive summary can be derived from the terms that are added to form a log-likelihood. In multi-state models if either of these exhaustive summaries are used then calculating the symbolic rank is not possible for $T > 3$; the computer will run out of memory trying to find the rank. Instead we use the life histories exhaustive summary to find a new exhaustive summary. We demonstrate how this is done using Model B as an example below.

Table 1: Matrices of probabilities used in the matrix notation for defining models AS, B and P. The symbol † refers to the dead state. Mo. is an abbreviation of model. In model AS $\phi_{i\dagger}^{(t)} = 1 - \sum_{j=1}^{N} \phi_{ij}^{(t)}$. In model B $\phi_{\star i\dagger}^{(t)} = 1 - \sum_{j=1}^{N} \phi_{\star ij}^{(t)}$. In models B and P, $\phi_{ij\dagger}^{(t)} = 1 - \sum_{k=1}^{N} \phi_{ijk}^{(t)}$. In addition, $\bar{p} = 1 - p$ and $\mathbf{\Pi}'$ represents the transpose of $\mathbf{\Pi}$.

Mo.	Initial state	Transition	Event
AS	$\mathbf{\Pi}'_t = \begin{bmatrix} \pi_1^{(t)} \\ \pi_2^{(t)} \\ \vdots \\ \pi_N^{(t)} \\ 0 \end{bmatrix}$	$\mathbf{\Phi}_t = \begin{bmatrix} \phi_{11}^{(t)} & \phi_{12}^{(t)} & \dots & \phi_{1N}^{(t)} & \phi_{1\dagger}^{(t)} \\ \phi_{21}^{(t)} & \phi_{22}^{(t)} & \dots & \phi_{2N}^{(t)} & \phi_{2\dagger}^{(t)} \\ \vdots & \vdots & & \vdots & \vdots \\ \phi_{N1}^{(t)} & \phi_{N2}^{(t)} & \dots & \phi_{NN}^{(t)} & \phi_{N\dagger}^{(t)} \\ 0 & 0 & \dots & 0 & 1 \end{bmatrix}$	$\mathbf{B}_t^0 = \begin{bmatrix} 0 & 0 & \dots & 0 & 1 \\ 1 & 0 & \dots & 0 & 0 \\ 0 & 1 & & 0 & 0 \\ & & \ddots & & \vdots \\ 0 & 0 & \dots & 1 & 0 \end{bmatrix}$ $\mathbf{B}_t = \begin{bmatrix} \bar{p}_1^{(t)} & \bar{p}_2^{(t)} & \dots & \bar{p}_N^{(t)} & 1 \\ p_1^{(t)} & 0 & \dots & 0 & 0 \\ 0 & p_2^{(t)} & & 0 & 0 \\ & & \ddots & & \vdots \\ 0 & 0 & \dots & p_N^{(t)} & 0 \end{bmatrix}$
B	$\mathbf{\Pi}'_t = \begin{bmatrix} \pi_1^{(t)} \\ \pi_2^{(t)} \\ \vdots \\ \pi_N^{(t)} \\ 0 \end{bmatrix}$	$\mathbf{\Phi}_t^0 = \begin{bmatrix} \phi_{\star 11}^{(t)} & \dots & \phi_{\star 1N}^{(t)} & \dots & 0 & \dots & 0 & \phi_{\star 1\dagger}^{(t)} \\ & & & \ddots & & & & \vdots \\ 0 & \dots & 0 & & \phi_{\star N1}^{(t)} & \dots & \phi_{\star NN}^{(t)} & \phi_{\star N\dagger}^{(t)} \\ 0 & \dots & 0 & \dots & 0 & \dots & 0 & 1 \end{bmatrix}$ $\mathbf{\Phi}_t = \begin{bmatrix} \phi_{111}^{(t)} & \dots & \phi_{11N}^{(t)} & \dots & 0 & \dots & 0 & \phi_{11\dagger}^{(t)} \\ & & & \ddots & & & & \vdots \\ 0 & \dots & 0 & & \phi_{1N1}^{(t)} & \dots & \phi_{1NN}^{(t)} & \phi_{1N\dagger}^{(t)} \\ \vdots & & & & & & & \vdots \\ \phi_{N11}^{(t)} & \dots & \phi_{N1N}^{(t)} & \dots & 0 & \dots & 0 & \phi_{N1\dagger}^{(t)} \\ & & & \ddots & & & & \vdots \\ 0 & \dots & 0 & & \phi_{NN1}^{(t)} & \dots & \phi_{NNN}^{(t)} & \phi_{NN\dagger}^{(t)} \\ 0 & \dots & 0 & \dots & 0 & \dots & 0 & 1 \end{bmatrix}$	$\mathbf{B}_t^0 = \begin{bmatrix} 0 & 0 & \dots & 0 & 1 \\ 1 & 0 & \dots & 0 & 0 \\ 0 & 1 & & 0 & 0 \\ & & \ddots & & \vdots \\ 0 & 0 & \dots & 1 & 0 \end{bmatrix}$ $\mathbf{B}'_t = \begin{bmatrix} \bar{p}_1^{(t)} & p_1^{(t)} & 0 \\ \vdots & \ddots & \\ \bar{p}_N^{(t)} & 0 & p_N^{(t)} \\ \vdots & & \vdots \\ \bar{p}_1^{(t)} & p_1^{(t)} & 0 \\ \vdots & \ddots & \\ \bar{p}_N^{(t)} & 0 & p_N^{(t)} \end{bmatrix}$
P	$\mathbf{\Pi}'_t = \begin{bmatrix} \pi_{11}^{(t)} \\ \vdots \\ \pi_{1N}^{(t)} \\ \vdots \\ \pi_{N1}^{(t)} \\ \vdots \\ \pi_{NN}^{(t)} \\ 0 \end{bmatrix}$	$\mathbf{\Phi}_t = \begin{bmatrix} \phi_{111}^{(t)} & \dots & \phi_{11N}^{(t)} & \dots & 0 & \dots & 0 & \phi_{11\dagger}^{(t)} \\ & & & \ddots & & & & \vdots \\ 0 & \dots & 0 & & \phi_{1N1}^{(t)} & \dots & \phi_{1NN}^{(t)} & \phi_{1N\dagger}^{(t)} \\ \vdots & & & & & & & \vdots \\ \phi_{N11}^{(t)} & \dots & \phi_{N1N}^{(t)} & \dots & 0 & \dots & 0 & \phi_{N1\dagger}^{(t)} \\ & & & \ddots & & & & \vdots \\ 0 & \dots & 0 & & \phi_{NN1}^{(t)} & \dots & \phi_{NNN}^{(t)} & \phi_{NN\dagger}^{(t)} \\ 0 & \dots & 0 & \dots & 0 & \dots & 0 & 1 \end{bmatrix}$	$(\mathbf{B}_t^0)' = \begin{bmatrix} 0 & 1 & 0 \\ \vdots & \ddots & \\ 0 & 0 & 1 \\ \vdots & & \vdots \\ 0 & 1 & 0 \\ \vdots & \ddots & \\ 0 & 0 & 1 \end{bmatrix}$ $\mathbf{B}'_t = \begin{bmatrix} \bar{p}_1^{(t)} & p_1^{(t)} & 0 \\ \vdots & \ddots & \\ \bar{p}_N^{(t)} & 0 & p_N^{(t)} \\ \vdots & & \vdots \\ \bar{p}_1^{(t)} & p_1^{(t)} & 0 \\ \vdots & \ddots & \\ \bar{p}_N^{(t)} & 0 & p_N^{(t)} \end{bmatrix}$

3.1 Model B

A method of finding a new simpler exhaustive summary, developed in Cole et al (2010), involves reparameterisation. We start by considering $N = 2$ states and $T = 2$. The exhaustive summary consisting of the probabilities of each possible history is

$$\boldsymbol{\kappa}(\boldsymbol{\theta}) = \begin{bmatrix} \text{Prob}(10) \\ \text{Prob}(20) \\ \text{Prob}(01) \\ \text{Prob}(11) \\ \text{Prob}(21) \\ \text{Prob}(02) \\ \text{Prob}(12) \\ \text{Prob}(22) \end{bmatrix} = \begin{bmatrix} \pi_1^{(1)} \left\{ \phi^{(1)}_{\star 11}(1 - p_1^{(2)}) + \phi^{(1)}_{\star 12}(1 - p_2^{(2)}) + (1 - \phi^{(1)}_{\star 11} - \phi^{(1)}_{\star 12}) \right\} \\ (1 - \pi_1^{(1)}) \left\{ \phi^{(1)}_{\star 21}(1 - p_1^{(2)}) + \phi^{(1)}_{\star 22}(1 - p_2^{(2)}) + (1 - \phi^{(1)}_{\star 11} - \phi^{(1)}_{\star 12}) \right\} \\ \pi_1^{(2)} \\ \pi_1^{(1)} \phi^{(1)}_{\star 11} p_1^{(2)} \\ (1 - \pi_1^{(1)}) \phi^{(1)}_{\star 21} p_1^{(2)} \\ 1 - \pi_1^{(2)} \\ \pi_1^{(1)} \phi^{(1)}_{\star 12} p_2^{(2)} \\ (1 - \pi_1^{(1)}) \phi^{(1)}_{\star 22} p_2^{(2)} \end{bmatrix},$$

which has parameter vector

$$\boldsymbol{\theta} = [\pi_1^{(1)}, \pi_1^{(2)}, \phi^{(1)}_{\star 11}, \phi^{(1)}_{\star 12}, \phi^{(1)}_{\star 21}, \phi^{(1)}_{\star 22}, p_1^{(2)}, p_2^{(2)}].$$

A reparameterisation, which is a set of parameters found together in $\boldsymbol{\kappa}(\boldsymbol{\theta})$, consists of the terms

$$\mathbf{s} = \begin{bmatrix} s_1 \\ s_2 \\ s_3 \\ s_4 \\ s_5 \\ s_6 \end{bmatrix} = \begin{bmatrix} \pi_1^{(1)} \\ \pi_1^{(1)} \phi^{(1)}_{\star 11} p_1^{(2)} \\ \pi_1^{(1)} \phi^{(1)}_{\star 12} p_2^{(2)} \\ (1 - \pi_1^{(1)}) \phi^{(1)}_{\star 21} p_1^{(2)} \\ (1 - \pi_1^{(1)}) \phi^{(1)}_{\star 22} p_2^{(2)} \\ \pi_1^{(2)} \end{bmatrix}.$$

We can rewrite $\boldsymbol{\kappa}(\boldsymbol{\theta})$ as a function of $\mathbf{s}$ which gives

$$\boldsymbol{\kappa}(\mathbf{s}) = \begin{bmatrix} s_1 - s_2 - s_3 \\ 1 - s_1 - s_4 - s_5 \\ s_6 \\ s_2 \\ s_4 \\ 1 - s_6 \\ s_3 \\ s_5 \end{bmatrix},$$

The derivative matrix

$$\mathbf{D}_s = \frac{\partial \boldsymbol{\kappa}(\mathbf{s})}{\partial \mathbf{s}} = \begin{bmatrix} 1 & -1 & 0 & 0 & 0 & 0 & 0 & 0 \\ -1 & 0 & 0 & 1 & 0 & 0 & 0 & 0 \\ -1 & 0 & 0 & 0 & 0 & 0 & 1 & 0 \\ 0 & -1 & 0 & 0 & 1 & 0 & 0 & 0 \\ 0 & -1 & 0 & 0 & 0 & 0 & 0 & 1 \\ 0 & 0 & 1 & 0 & 0 & -1 & 0 & 0 \end{bmatrix}$$

has rank 6. As the rank is equal to the number of reparameterisation terms then we say that $\mathbf{D}_s$ is full rank. If $\mathbf{D}_s$ is full rank then the reparameterisation $\mathbf{s}$ is a new exhaustive summary (Cole et al, 2010).

We then extend to $T = 3$. We relabel the terms in $\mathbf{s}$ to enable the use of the extension theorem to generalise the result. The reparameterisation then consists of the terms

$$\mathbf{s} = \begin{bmatrix} s_1 \\ s_2 \\ s_3 \\ s_4 \\ s_5 \\ s_6 \\ s_7 \\ s_8 \\ s_9 \\ s_{10} \\ s_{11} \\ s_{12} \\ s_{13} \\ s_{14} \\ s_{15} \\ s_{16} \\ s_{17} \\ s_{18} \\ s_{19} \\ s_{20} \\ s_{21} \end{bmatrix} = \begin{bmatrix} \pi_1^{(2)} \\ \pi_1^{(2)}\phi_{\star 11}^{(2)}p_1^{(3)} \\ \pi_1^{(2)}\phi_{\star 12}^{(2)}p_2^{(3)} \\ (1-\pi_1^{(2)})\phi_{\star 21}^{(2)}p_1^{(3)} \\ (1-\pi_1^{(2)})\phi_{\star 22}^{(2)}p_2^{(3)} \\ \pi_1^{(3)} \\ \pi_1^{(1)} \\ \pi_1^{(1)}\phi_{\star 11}^{(1)}p_1^{(2)} \\ \pi_1^{(1)}\phi_{\star 12}^{(1)}p_2^{(2)} \\ (1-\pi_1^{(1)})\phi_{\star 21}^{(1)}p_1^{(2)} \\ (1-\pi_1^{(1)})\phi_{\star 22}^{(1)}p_2^{(2)} \\ \phi_{111}^{(2)}p_1^{(3)} \\ \phi_{112}^{(2)}p_2^{(3)} \\ \phi_{121}^{(2)}p_1^{(3)} \\ \phi_{122}^{(2)}p_2^{(3)} \\ \phi_{211}^{(2)}p_1^{(3)} \\ \phi_{212}^{(2)}p_2^{(3)} \\ \phi_{221}^{(2)}p_1^{(3)} \\ \phi_{222}^{(2)}p_2^{(3)} \\ p_1^{(2)} \\ p_2^{(2)} \end{bmatrix}.$$

The exhaustive summary terms

$$\boldsymbol{\kappa}_1(\mathbf{s}_{p1}) = \begin{bmatrix} \text{Prob}(010) \\ \text{Prob}(020) \\ \text{Prob}(001) \\ \text{Prob}(011) \\ \text{Prob}(021) \\ \text{Prob}(002) \\ \text{Prob}(012) \\ \text{Prob}(022) \end{bmatrix} = \begin{bmatrix} s_1 - s_2 - s_3 \\ 1 - s_1 - s_4 - s_5 \\ s_6 \\ s_2 \\ s_4 \\ 1 - s_6 \\ s_3 \\ s_5 \end{bmatrix},$$

are then equivalent to the terms when $T = 2$, with $\mathbf{s}_{p1} = [s_1, \ldots, s_6]$. All the additional terms go in the vector

$$\boldsymbol{\kappa}_2(\mathbf{s}) = \begin{bmatrix} \text{Prob}(100) \\ \vdots \\ \text{Prob}(222) \end{bmatrix} = \begin{bmatrix} s_9 s_{14} + s_8 s_{12} - s_8 + \ldots s_9 s_{15} \\ \vdots \\ s_{11} s_{19} \end{bmatrix}$$

with extra s_i parameters $\mathbf{s}_{p2} = [s_7, \ldots, s_{21}]$ We know that $\mathbf{D}_{s,1} = \partial \boldsymbol{\kappa}_1(\mathbf{s}_{p1})/\partial \mathbf{s}_{p1}$ has full rank 6. We can also show that $\mathbf{D}_{s,2} = \partial \boldsymbol{\kappa}_2(\mathbf{s})/\partial \mathbf{s}_{p2}$ has full rank 15. By the extension theorem of Catchpole and Morgan (1997) if $\mathbf{D}_{s,1}$ and $\mathbf{D}_{s,2}$ are full rank then the matrix $\mathbf{D}_s$ must be full rank. As we will always add the same addition terms each year, and using a similar extension theorem for increasing the number of states, it follows that a new exhaustive summary consists of the terms

$$\boldsymbol{\kappa} = \begin{bmatrix} \pi_i^{(t)} & t = 1, \ldots, T, i = 1, \ldots, N-1 \\ \pi_i^{(t)} \phi_{\star ij}^{(t)} p_j^{(t+1)} & t = 1, \ldots, T-2, i = 1, \ldots, N-1, j = 1, \ldots, N \\ \left(1 - \sum_{k=1}^{N-1} \pi_k^{(t)}\right) \phi_{\star ij}^{(t)} p_j^{(t+1)} & t = 1, \ldots, T-1, i = N, j = 1, \ldots, N \\ \phi_{ijk}^{(t)} p_k^{(t+1)} & t = 2, \ldots, T-1, i, j, k = 1, \ldots, N \\ p_i^{(t)} & t = 2, \ldots, T-1, i = 1, \ldots, N \end{bmatrix}.$$

3.2 Model AS

Using a similar method we can derive a simpler exhaustive summary for model AS. This consists of the probability combinations:

$$\boldsymbol{\kappa} = \begin{bmatrix} \pi_i^{(t)} & \text{for } t = 1, \ldots, T, i = 1, \ldots, N-1 \\ \pi_i^{(t)} \phi_{ij}^{(t)} p_j^{(t+1)} & \text{for } t = 1, \ldots, T-1, i = 1, \ldots, N-1, j = 1, \ldots, N \\ \left(1 - \sum_{k=1}^{N-1} \pi_k^{(t)}\right) \phi_{ij}^{(t)} p_j^{(t+1)} & \text{for } t = 1, \ldots, T-1, i = N, j = 1, \ldots, N \\ p_i^{(t)} & \text{for } t = 2, \ldots, T-1, i = 1, \ldots, N \end{bmatrix}. \quad (1)$$

We demonstrate how to use equations (1) to investigate parameter redundancy with an example where there are $N = 2$ sites, and the capture probabilities are not dependent on the capture occasion. For $T = 3$ occasions the probability combinations are

$$\begin{aligned} \boldsymbol{\kappa}' = & \left[\pi_1^{(1)}, \pi_1^{(2)}, \pi_1^{(3)}, \pi_1^{(1)} \phi_{11}^{(1)} p_1, \pi_1^{(1)} \phi_{12}^{(1)} p_2, \pi_1^{(2)} \phi_{11}^{(2)} p_1, \pi_1^{(2)} \phi_{12}^{(2)} p_2, (1 - \pi_1^{(1)}) \phi_{21}^{(1)} p_1, (1 - \pi_1^{(1)}) \phi_{22}^{(1)} p_2, \right. \\ & \left. (1 - \pi_1^{(2)}) \phi_{21}^{(2)} p_1, (1 - \pi_1^{(2)}) \phi_{22}^{(2)} p_2, p_1, p_2 \right]. \end{aligned}$$

The parameters are

$$\boldsymbol{\theta} = [\pi_1^{(1)}, \pi_1^{(2)}, \pi_1^{(3)}, \phi_{11}^{(1)}, \phi_{11}^{(2)}, \phi_{12}^{(1)}, \phi_{12}^{(2)}, \phi_{21}^{(1)}, \phi_{21}^{(2)}, \phi_{22}^{(1)}, \phi_{22}^{(2)}, p_1, p_2,],$$

The terms are of this derivative matrix are

$$\mathbf{D}_1 = \frac{\partial \boldsymbol{\kappa}}{\partial \boldsymbol{\theta}} = \begin{bmatrix} 1 & 0 & 0 & \phi_{11}^{(1)} & \ldots \\ 0 & 1 & 0 & 0 & \ldots \\ 0 & 0 & 1 & 0 & \ldots \\ 0 & 0 & 0 & \pi_1^{(1)} p_1 & \ldots \\ \vdots & & & & \end{bmatrix}.$$

The derivative matrix, $\mathbf{D}_1$, has rank 13, so that the model has deficiency $d = 0$ and is not parameter redundant.

It is also possible to generalise a result to any number of occasions. In this example adding an extra occasion so that $T = 4$ extends the probability combinations, the new additional terms are

$$\boldsymbol{\kappa}'_{ex} = \left[\pi_1^{(4)}, \pi_1^{(3)}\phi_{11}^{(3)}p_1, \pi_1^{(3)}\phi_{12}^{(3)}p_2, (1-\pi_1^{(3)})\phi_{21}^{(3)}p_1, (1-\pi_1^{(3)})\phi_{22}^{(3)}p_2\right].$$

and there are also 5 new parameters introduced: $\boldsymbol{\theta}_{ex} = [\pi_1^{(4)}, \phi_{11}^{(3)}, \phi_{12}^{(3)}, \phi_{21}^{(3)}, \phi_{22}^{(3)}]$. Rather than considering the complete derivative matrix we only need to consider the part corresponding to the additional terms and parameters, which is

$$\mathbf{D}_{ex} = \frac{\partial \boldsymbol{\kappa}_{ex}}{\partial \boldsymbol{\theta}_{ex}} = \begin{bmatrix} 1 & 0 & 0 & 0 & 0 \\ 0 & \pi_1^{(3)}p_1 & 0 & 0 & 0 \\ 0 & 0 & \pi_1^{(3)}p_2 & 0 & 0 \\ 0 & 0 & 0 & (1-\pi_1^{(3)})p_1 & 0 \\ 0 & 0 & 0 & 0 & (1-\pi_1^{(3)})p_2 \end{bmatrix}.$$

As the rank of $\mathbf{D}_{ex}$ is 5, the maximum rank that a matrix of that size can obtain, we can use the extension theorem of Catchpole and Morgan (1997) to conclude that for $T \geq 3$ the model is not parameter redundant. We can apply a similar extension theorem for increasing the number of sites. For $N \geq 2$ sites and $T \geq N$ occasions, the rank is $N^2T - N^2 + NT - T$, and there are $N^2T - N^2 + NT - T$ parameters; therefore the model will always have deficiency $d = 0$.

3.3 Model P

A similar exhaustive summary can also be derived for model P. For Model P the probability combinations are:

$$\boldsymbol{\kappa} = \begin{bmatrix} \sum_{i=1}^{N} \pi_{ij}^{(t)} & t = 1,\ldots,T, j = 1,\ldots,N-1 \\ \pi_{ij}^{(t)} & t = 2,\ldots,T-1, i = 2,\ldots,N, j = 1,\ldots,N-1, \text{ and } i = 1,\ldots,N-1, j = N \\ \sum_{i=1}^{N} \pi_{ij}^{(1)}\phi_{ijk}^{(1)}p_k^{(2)} & j,k = 1,\ldots,N \\ \phi_{ijk}^{(t)}p_k^{(t+1)} & t = 2,\ldots,T-1, i,j,k = 1,\ldots,N, \\ p_i^{(t)} & t = 2,\ldots,T-1, i = 1,\ldots,N \end{bmatrix}. \quad (2)$$

An example of checking parameter redundancy in model P using equation (2) is as follows. We suppose that there are $N = 2$ sites and the recapture probability and initial state probability do not depend the capture occasion, but the transition probabilities do depend on capture occasion.

For $T = 3$ occasions the exhaustive summary is

$$\boldsymbol{\kappa}_{1,\ldots,9} = \begin{bmatrix} \pi_{11}+\pi_{21} \\ \pi_{21} \\ \pi_{22} \\ \pi_{11}\phi^{(1)}_{111}p_1 + \phi_{21}\phi^{(1)}_{211}p_1 \\ \pi_{11}\phi^{(1)}_{112}p_2 + \pi_{21}\phi^{(1)}_{212}p_2 \\ \pi_{12}\phi^{(1)}_{121}p_1 + (1-\pi_{11}-\pi_{12}-\pi_{21})\phi^{(1)}_{221}p_1 \\ (1-\pi_{11}-\pi_{12}-\pi_{21})\pi_{12}\phi^{(1)}_{122}p_2 + \phi^{(1)}_{222}p_2 \\ \phi^{(2)}_{111}p_1 \\ \phi^{(2)}_{112}p_2 \end{bmatrix} \quad \boldsymbol{\kappa}_{10,\ldots,17} = \begin{bmatrix} \phi^{(2)}_{121}p_1 \\ \phi^{(2)}_{122}p_2 \\ \phi^{(2)}_{211}p_1 \\ \phi^{(2)}_{212}p_2 \\ \phi^{(2)}_{221}p_1 \\ \phi^{(2)}_{222}p_2 \\ p_1 \\ p_2 \end{bmatrix}.$$

The derivative matrix, formed by differentiating $\boldsymbol{\kappa}$ with respect to the parameters

$$\boldsymbol{\theta} = [\pi_{11}, \pi_{21}, \pi_{12}, \phi^{(1)}_{111}, \ldots, \phi^{(2)}_{222}, p_1, p_2],$$

has rank 17. There are 21 parameters in the model, so that this model is parameter redundant with deficiency $d = 4$. Solving the appropriate set of partial differential equations, the estimable parameters and parameter combinations are π_{11}, π_{21}, π_{12}, $\phi^{(2)}_{111}, \ldots, \phi^{(2)}_{222}$, p_1, p_2, $\gamma_1 = \{(1-\pi_{11}-\pi_{12}-\pi_{21})\phi^{(1)}_{2,2,2} - \pi_{12}\phi^{(1)}_{122}\}/(1-\pi_{11}-\pi_{12}-\pi_{21})$, $\gamma_2 = \{(1-\pi_{11}-\pi_{12}-\pi_{21})\phi^{(1)}_{2,2,1} - \pi_{12}\phi^{(1)}_{121}\}/(1-\pi_{11}-\pi_{12}-\pi_{21})$, $\gamma_3 = (\pi_{11}\phi^{(1)}_{112} + \pi_{21}\phi^{(1)}_{212})/\pi_{21}$ and $\gamma_4 = (\pi_{11}\phi^{(1)}_{111} + \pi_{21}\phi^{(1)}_{211})/\pi_{21}$.

To use the extension theorem with parameter redundant models we first need to reparameterise the model so that the rank is the same as the number of parameters. The extension theorem can be applied to the reparameterised model. The rank of the reparameterised model will be the same as the rank of the original parameterisation (Cole et al, 2010). We use the estimable parameter combinations as the new set of parameters. The exhaustive summary is then

$$\boldsymbol{\kappa}_{re,1,\ldots,9} = \begin{bmatrix} \pi_{11}+\pi_{21} \\ \pi_{21} \\ \pi_{22} \\ \pi_{21}p_1\gamma_4 \\ \pi_{21}p_2\gamma_3 \\ (1-\pi_{11}-\pi_{12}-\pi_{21})p_1\gamma_2 \\ (1-\pi_{11}-\pi_{12}-\pi_{21})p_2\gamma_1 \\ \phi^{(2)}_{111}p_1 \\ \phi^{(2)}_{112}p_2 \end{bmatrix} \quad \boldsymbol{\kappa}_{re,10,\ldots,17} = \begin{bmatrix} \phi^{(2)}_{121}p_1 \\ \phi^{(2)}_{122}p_2 \\ \phi^{(2)}_{211}p_1 \\ \phi^{(2)}_{212}p_2 \\ \phi^{(2)}_{221}p_1 \\ \phi^{(2)}_{222}p_2 \\ p_1 \\ p_2 \end{bmatrix}$$

and the 17 parameters are

$$\boldsymbol{\theta}_{re} = [\pi_{11}, \pi_{21}, \pi_{12}, \phi^{(2)}_{111}, \ldots, \phi^{(2)}_{222}, \gamma_1, \ldots, \gamma_4, p_1, p_2].$$

We can then apply the extension theorem to this reparameterised model and show that it always has rank $8T - 7$. By the reparameterisation theorem of Cole et al (2010) the original model also has rank $8T - 7$ but there are $8T - 3$ parameters so the model will always have deficiency $d = 4$ for any $T \geq 3$.

Note that for model P equation (2) is not valid if $\phi_{ijk}^{(t)} = \phi_{jk}^{(t)}$ for all i and for any j, k or t. This occurs when there is no memory in the model, so that typically model AS would be used in this case. However we do need to consider model P with no memory when performing a score test. An exhaustive summary for model P when $\phi_{ijk}^{(t)} = \phi_{jk}^{(t)}$ for all i, j, k and t consists of the terms:

$$\boldsymbol{\kappa} = \begin{bmatrix} \sum_{i=1}^{N} \pi_{ij}^{(t)} & t = 1, \ldots, T, j = 1, \ldots, N-1 \\ \phi_{1jk}^{(1)} p_k^{(2)} \sum_{i=1}^{N} \pi_{ij}^{(1)} & j = 1, \ldots, N, k = 1, \ldots, N \\ \phi_{1jk}^{(t)} p_k^{(t+1)} & t = 2, \ldots, T-1, j = 1, \ldots, N, k = 1, \ldots, N \\ p_i^{(t)} & t = 2, \ldots, T-1, i = 1, \ldots, N \end{bmatrix}. \tag{3}$$

The model we use for the score tests under model P has transition matrix

$$\boldsymbol{\Phi}_t = \begin{bmatrix} \phi_{111}^{(t)} & \phi_{112}^{(t)} & 0 & 0 & \phi_{11\dagger}^{(t)} \\ 0 & 0 & \phi_{121}^{(t)} & \phi_{122}^{(t)} & \phi_{12\dagger}^{(t)} \\ \phi_{211}^{(t)} & \phi_{212}^{(t)} & 0 & 0 & \phi_{21\dagger}^{(t)} \\ 0 & 0 & \phi_{221}^{(t)} & \phi_{222}^{(t)} & \phi_{22\dagger}^{(t)} \\ 0 & 0 & 0 & 0 & 1 \end{bmatrix} = \begin{bmatrix} s\psi_{111} & s(1-\psi_{111}) & 0 & 0 & 1-s \\ 0 & 0 & s(1-\psi_{122}) & s\psi_{122} & 1-s \\ s\psi_{211} & s(1-\psi_{211}) & 0 & 0 & 1-s \\ 0 & 0 & s(1-\psi_{222}) & s\psi_{222} & 1-s \\ 0 & 0 & 0 & 0 & 1 \end{bmatrix}, \tag{4}$$

where s is the probability of surviving from one occasion to the next and ψ_{ijj} is the probability an animal stays at the same site j given that it was at site i on the previous occasion. Under this model when $T = 3$ and $N = 2$ and $\psi_{ijk} = \psi_{jk}$ for all i, j and k, the exhaustive summary is

$$\boldsymbol{\kappa} = \begin{bmatrix} \pi_{11} + \pi_{21} \\ \pi_{11} s\psi_{11} p_1 + \pi_{21} s\psi_{11} p_1 \\ \pi_{11} s\bar{\psi}_{11} p_2 + \pi_{21} s\bar{\psi}_{11} p_2 \\ \pi_{12} s\bar{\psi}_{22} p_1 + (1 - \pi_{11} - \pi_{12} - \pi_{21}) s\bar{\psi}_{22} p_1 \\ \pi_{12} s\psi_{22} p_2 + (1 - \pi_{11} - \pi_{12} - \pi_{21}) s\psi_{22} p_2 \\ s\psi_{11} p_1 \\ s\bar{\psi}_{11} p_2 \\ s\bar{\psi}_{22} p_1 \\ s\psi_{22} p_2 \\ p_1 \\ p_2 \end{bmatrix} = \begin{bmatrix} \pi_{11} + \pi_{21} \\ \pi_{11} s\psi_{11} p_1 + \pi_{21} s\psi_{11} p_1 \\ \pi_{11} s\bar{\psi}_{11} p_2 + \pi_{21} s\bar{\psi}_{11} p_2 \\ (1 - \pi_{11} - \pi_{21}) s\bar{\psi}_{22} p_1 \\ (1 - \pi_{11} - \pi_{21}) s\psi_{22} p_2 \\ s\psi_{11} p_1 \\ s\bar{\psi}_{11} p_2 \\ s\bar{\psi}_{22} p_1 \\ s\psi_{22} p_2 \\ p_1 \\ p_2 \end{bmatrix},$$

where $\bar{\psi}_{ii} = 1 - \psi_{ii}$. Note that because $\pi_{22} = 1 - \pi_{11} - \pi_{12} - \pi_{21}$ the parameter π_{12} does not appear at all in the exhaustive summary above. However we need to consider putting constraints on the π_{ij} so that $\pi_{11} = \pi_{21}$ and $\pi_{12} = \pi_{22}$ with $\pi_{22} = 1 - \pi_{11} - \pi_{12} - \pi_{21}$. To be able to use the score test we use the additional constraint $\pi_{12} = \frac{1}{2} - \pi_{11}$ (as this is the solution of $2\pi_{11} + 2\pi_{12} = 1$.

3.4 Alternative Parameterisations

It is not necessary to use the same parameterisation as described here. The same methods can be used to check for parameter redundancy for any alternative parameterisation. This is due to

the reparameterisation theorem of Cole et al (2010). One such example involves separating out survival and transition probabilities in the transition matrix. This is the same parameterisation used in the simulated data sets. The transition matrix is given by equation (4). Using equation (2) when $N = 2$ and $T = 3$, the parameter combinations to check for parameter redundancy are then

$$\boldsymbol{\kappa}' = [\pi_{11} + \pi_{21}, \pi_{12}, \pi_{21}, \pi_{11} s \psi_{111} p_1 + \pi_{21} s \psi_{211} p_1, \ldots, p_1, p_2] \,.$$

The 10 parameters are $\boldsymbol{\theta} = [\pi_{11}, \pi_{12}, \pi_{21}, s, \psi_{111}, \psi_{122}, \psi_{211}, \psi_{222}, p_1, p_2]$. The derivative matrix $\mathbf{D} = \partial \boldsymbol{\kappa} / \partial \boldsymbol{\theta}$ has rank 10 so that the the model is not parameter redundant. Using the extension theorem we can generalise this result to any $T \geq 3$ and $N \geq 2$; this model will always have rank $N^3 + N$, so that in theory all of the $N^3 + N$ parameters can be estimated.

4 Diagnostic goodness of fit tests

4.1 Guide for executing the WBWA test

In this section we provide a guide that explains how to carry out a WBWA test in U-CARE (Choquet et al, 2009). There are three steps to execute in order to carry out a WBWA test in U-CARE.

1. Firstly the data need to be loaded into U-CARE. From the top file choose ‘File’ and then ‘Open (Mark Format)’, then select the data set.
2. Next there is the following question ‘How many individual covariates in the file?’ For this test choose 0.
3. Lastly the WBWA test is executed. From the top menu choose ‘GOODNESS-OF-FIT for Multistate’, and then choose the option ‘TEST WBWA’.

We demonstrate how the WBWA test works using two simulated data sets. The parameters used to simulate are the same as those described in the main paper. The U-CARE output for these two data sets is given in Figure 4.1 and the results are summarised in Table 2. The data set with memory has a p-value for the WBWA test of <0.01, therefore the conclusion of the test is that a memory model could be a suitable model, so model B or model P should be fitted. The data set without memory has a p-value for the WBWA test of 0.60, suggesting that a memory model is not needed for this data set, so that model AS should be fitted.

4.2 Further simulation for N=3 sites

We extend the simulation used in the paper to three sites. We also consider two instances of a three-site model: when there is memory (M) and when there is not memory ($\bar{M}$). The simulated

Figure 1: U-CARE output for the WBWA test for the example with memory. The last line gives the WBWA test, the p-value and the degrees of freedom.

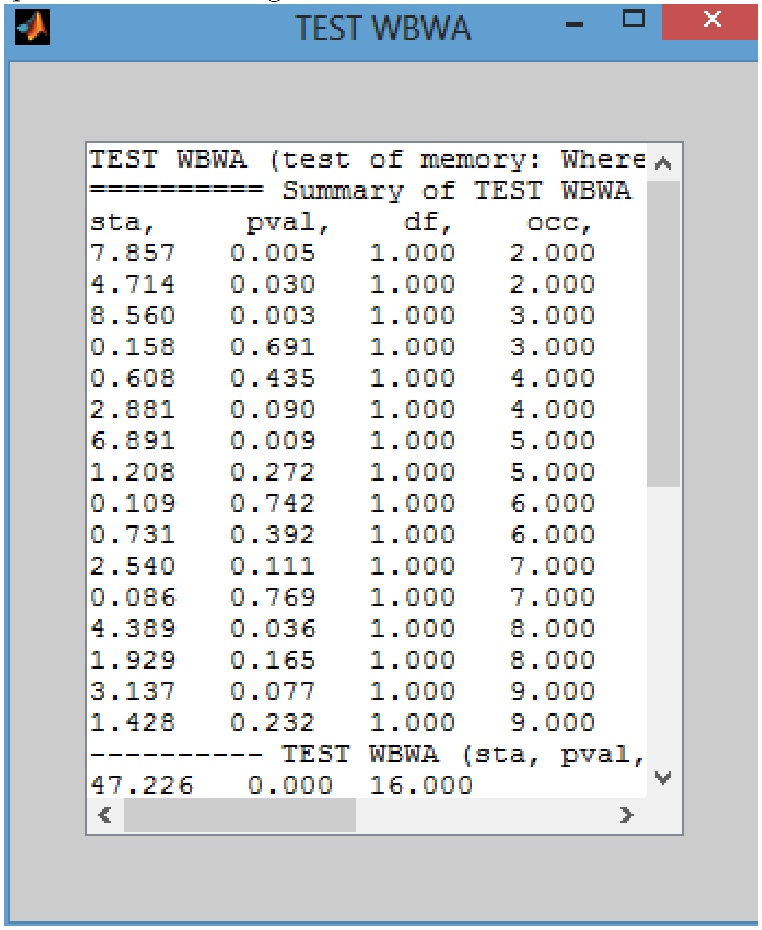

Table 2: WBWA results. M denotes the data set that has memory. $\bar{M}$ denotes the data set that has no memory.

	M	$\bar{M}$
WBWA test statistic	47.2	14.0
P-value	<0.01	0.60

data sets with memory have transition matrix

$$\begin{aligned}\mathbf{\Phi}_t &= \begin{bmatrix} \phi^{(t)}_{111} & \phi^{(t)}_{112} & \phi^{(t)}_{113} & 0 & 0 & 0 & 0 & 0 & 0 & \phi^{(t)}_{11\dagger} \\ 0 & 0 & 0 & \phi^{(t)}_{121} & \phi^{(t)}_{122} & \phi^{(t)}_{123} & 0 & 0 & 0 & \phi^{(t)}_{12\dagger} \\ 0 & 0 & 0 & 0 & 0 & 0 & \phi^{(t)}_{131} & \phi^{(t)}_{132} & \phi^{(t)}_{133} & \phi^{(t)}_{13\dagger} \\ \phi^{(t)}_{211} & \phi^{(t)}_{212} & \phi^{(t)}_{213} & 0 & 0 & 0 & 0 & 0 & 0 & \phi^{(t)}_{21\dagger} \\ 0 & 0 & 0 & \phi^{(t)}_{221} & \phi^{(t)}_{222} & \phi^{(t)}_{223} & 0 & 0 & 0 & \phi^{(t)}_{22\dagger} \\ 0 & 0 & 0 & 0 & 0 & 0 & \phi^{(t)}_{231} & \phi^{(t)}_{232} & \phi^{(t)}_{233} & \phi^{(t)}_{23\dagger} \\ \phi^{(t)}_{311} & \phi^{(t)}_{312} & \phi^{(t)}_{313} & 0 & 0 & 0 & 0 & 0 & 0 & \phi^{(t)}_{31\dagger} \\ 0 & 0 & 0 & \phi^{(t)}_{321} & \phi^{(t)}_{322} & \phi^{(t)}_{323} & 0 & 0 & 0 & \phi^{(t)}_{32\dagger} \\ 0 & 0 & 0 & 0 & 0 & 0 & \phi^{(t)}_{331} & \phi^{(t)}_{332} & \phi^{(t)}_{333} & \phi^{(t)}_{33\dagger} \\ 0 & 0 & 0 & 0 & 0 & 0 & 0 & 0 & 0 & 1 \end{bmatrix} \\ &= \begin{bmatrix} s\psi_{111} & s\psi_{112} & s\psi_{113} & 0 & 0 & 0 & 0 & 0 & 0 & 1-s \\ 0 & 0 & 0 & s\psi_{121} & s\psi^{(t)}_{122} & s\psi_{123} & 0 & 0 & 0 & 1-s \\ 0 & 0 & 0 & 0 & 0 & 0 & s\psi_{131} & s\psi_{132} & s\psi_{133} & 1-s \\ s\psi_{211} & s\psi_{212} & s\psi_{213} & 0 & 0 & 0 & 0 & 0 & 0 & 1-s \\ 0 & 0 & 0 & s\psi_{221} & s\psi_{222} & s\psi_{223} & 0 & 0 & 0 & 1-s \\ 0 & 0 & 0 & 0 & 0 & 0 & \psi_{231} & \psi_{232} & \psi_{233} & 1-s \\ s\psi_{311} & s\psi_{312} & s\psi_{313} & 0 & 0 & 0 & 0 & 0 & 0 & 1-s \\ 0 & 0 & 0 & s\psi_{321} & s\psi_{322} & s\psi_{323} & 0 & 0 & 0 & 1-s \\ 0 & 0 & 0 & 0 & 0 & 0 & s\phi_{331} & s\phi_{332} & s\phi_{333} & 1-s \\ 0 & 0 & 0 & 0 & 0 & 0 & 0 & 0 & 0 & 1 \end{bmatrix}, \end{aligned} \tag{5}$$

where s is the probability of surviving from one occasion to the next and ψ_{ijj} is the probability an animal stays at the same site j given that it was at site i on the previous occasion. For the simulation we set the parameters to $\pi_{11} = \pi_{12} = \pi_{13} = \ldots = \pi_{33} = 1/9$, $s = 0.9$, $\psi_{111} = 0.7$, $\psi_{111} = 0.7$, $\psi_{112} = 0.15$, $\psi_{113} = 0.15$ $\psi_{121} = 1/3$, $\psi_{122} = 1/3$, $\psi_{123} = 1/3$,$\psi_{131} = 1/3$, $\psi_{132} = 1/3$, $\psi_{133} = 1/3$, $\psi_{211} = 1/3$, $\psi_{212} = 1/3$, $\psi_{213} = 1/3$, $\psi_{221} = 0.2$, $\psi_{222} = 0.6$, $\psi_{223} = 0.2$, $\psi_{231} = 1/3$, $\psi_{232} = 1/3$, $\psi_{233} = 1/3$, $\psi_{311} = 1/3$, $\psi_{312} = 1/3$, $\psi_{313} = 1/3$,$\psi_{321} = 1/3$, $\psi_{322} = 1/3$, $\psi_{323} = 1/3$, $\psi_{331} = 0.1$, $\psi_{332} = 0.1$, $\psi_{333} = 0.8$, $p_1 = 0.5$, $p_2 = 0.3$ and $p_3 = 0.4$.

For the simulated data sets without memory the transition matrix is

$$\mathbf{\Phi}_t = \begin{bmatrix} \phi^{(t)}_{11} & \phi^{(t)}_{12} & \phi^{(t)}_{13} & \phi^{(t)}_{1\dagger} \\ \phi^{(t)}_{21} & \phi^{(t)}_{22} & \phi^{(t)}_{32} & \phi^{(t)}_{2\dagger} \\ \phi^{(t)}_{31} & \phi^{(t)}_{32} & \phi^{(t)}_{33} & \phi^{(t)}_{3\dagger} \\ 0 & 0 & 0 & 1 \end{bmatrix} = \begin{bmatrix} s\psi_{11} & s\psi_{11} & s\psi_{13} & 1-s \\ s\psi_{21} & s\psi_{21} & s\psi_{23} & 1-s \\ s\psi_{31} & s\psi_{31} & s\psi_{33} & 1-s \\ 0 & 0 & 0 & 1 \end{bmatrix}, \tag{6}$$

and the parameter values used are $\pi_1 = 0.5$, $s = 0.9$, $\psi_{11} = 0.7$, $\psi_{12} = 0.15$, $\psi_{13} = 0.15$, $\psi_{21} = 0.2$, $\psi_{22} = 0.6$, $\psi_{23} = 0.2$, $\psi_{31} = 0.1$, $\psi_{32} = 0.1$, $\psi_{33} = 7$, $p_1 = 0.5$, $p_2 = 0.3$ and $p_3 = 0.2$. As with the two-site simulation we suppose that there are $T = 10$ years of data and that $m = 25, 50, 75, 100, 125, 150$ animals are marked each year at each site, so that total sample sizes are 750, 1500, 2250, 3000, 3750 and 4500 respectively. For each value of m 100 data sets are simulated.

In Table 3 we present further statistics from the simulation study. We give the average p-value along with the standard deviation of the p-value.

Table 3: The average p-value along with the standard deviation of the p-value in brackets for the simulation study, split by whether the simulation had memory, (a) or did not have memory (b). In the simulation m is the number of animals marked per year per site and N is the number of sites. WBWA refers to the WBWA test. Score B refers a score test comparing model AS with model B. Score P refers to a score test comparing model AS with model P.

(a) Simulation with Memory

	$N = 2$			$N = 3$		
m	WBWA	Score B	Score P	WBWA	Score B	Score P
25	0.42 (0.32)	0.02 (0.11)	<0.01 (0.01)	0.44 (0.27)	0.11 (0.25)	0.05 (0.17)
50	0.17 (0.19)	<0.01 (<0.01)	<0.01 (<0.01)	0.12 (0.19)	0.03 (0.14)	0.03 (0.15)
75	0.07 (0.13)	<0.01 (<0.01)	<0.01 (<0.01)	0.06 (0.14)	<0.01 (0.07)	<0.01 (<0.01)
100	0.04 (0.10)	<0.01 (<0.01)	<0.01 (<0.01)	0.03 (0.08)	0.02 (0.11)	<0.01 (0.02)
125	0.01 (0.04)	<0.01 (<0.01)	<0.01 (<0.01)	<0.01 (0.02)	0.02 (0.12)	0.02 (0.11)
150	<0.01 (0.03)	<0.01 (<0.01)	<0.01 (<0.01)	<0.01 (0.02)	0.01 (0.33)	0.02 (0.13)

(b) Simulation without Memory

	N=2			N=3		
m	WBWA	Score B	Score P	WBWA	Score B	Score P
25	0.82 (0.20)	0.47 (0.31)	0.51 (0.32)	0.78 (0.23)	0.48 (0.39)	0.50 (0.36)
50	0.54 (0.29)	0.45 (0.29)	0.46 (0.30)	0.57 (0.31)	0.67 (0.34)	0.63 (0.36)
75	0.53 (0.29)	0.50 (0.32)	0.47 (0.28)	0.51 (0.27)	0.52 (0.35)	0.52 (0.31)
100	0.52 (0.26)	0.47 (0.29)	0.48 (0.28)	0.52 (0.30)	0.53 (0.31)	0.52 (0.30)
125	0.53 (0.27)	0.51 (0.27)	0.51 (0.28)	0.56 (0.29)	0.48 (0.28)	0.53 (0.28)
150	0.49 (0.29)	0.48 (0.29)	0.46 (0.27)	0.45 (0.27)	0.49 (0.33)	0.48 (0.30)

Table 4: Comparison of Score test (Score) results with a likelihood-ratio test (LR) and AIC model selection. M denotes the data set that has memory. $\bar{M}$ denotes the data set that has no memory. In each case the alternative hypothesis (H_1) is either model B or model P.

H_1: Model B:

	Score	DF	P-value	LR	DF	P-value	AIC AS	AIC B
M	133.63	4	<0.01	142.69	4	<0.01	20406	**20271**
$\bar{M}$	3.80	4	0.43	3.82	4	0.42	**19624**	19628

H_1: Model P:

	Score	DF	P-value	LR	DF	P-value	AIC AS	AIC P
M	130.24	2	<0.01	142.07	2	<0.01	20406	**20271**
$\bar{M}$	1.70	2	0.42	1.75	2	0.43	**19624**	19630

5 Score Tests

We demonstrate how the score tests work using the same two simulated data sets as the WBWA test. We also compare score tests with likelihood ratio tests and AIC for model selection, in Table 4. The score test, likelihood0ratio tests and the AIC model selection all produce the same results. For the first simulation a memory model is recommended. For the second simulation model AS is sufficient. We note that to perform the score test we only need to fit model AS, which forms the null hypothesis, but to perform the likelihood-ratio test and find the AIC we need to fit model AS and models B or P, as appropriate.

6 Guide for Model fitting using E-surge

In this section we demonstrate how memory models can be fitted in E-SURGE. Here we examine how to fit model P in which the survival-transition probability is further subdivided into the probability of survival and the probability of movement from each site to the others conditional on surviving. This formulation is the analogue of the separate formulation of the conditional Arnason-Schwarz model.

With the 2 geographical sites 1 and 2, the states are {'11', '12', '21', '22', '†'}. State '1' denotes presence at site 1 at time t-1 and t, state '12' denotes presence at site 1 at time $t-1$ and presence at site 2 at time t, and so on; the '†' state refers to the dead state. Assuming that if an animal is seen and its current site is known without error, the set of events (i.e., the results of observations) is

$$\Omega = \{\text{'not seen', 'seen at 1', 'seen at 2'}\}.$$

We assumed that the model is homogeneous in time (i.e. not time dependent). The initial state matrix is

$$\mathbf{\Pi} = \begin{bmatrix} \pi_{11} & \pi_{12} & \pi_{21} & \pi_{22} & 0 \end{bmatrix}.$$

We consider a model defined by two elementary transition matrices,

$$\mathbf{S} = \begin{bmatrix} s_{11} & 0 & 0 & 0 & 1-s_{11} \\ 0 & s_{12} & 0 & 0 & 1-s_{12} \\ 0 & 0 & s_{21} & 0 & 1-s_{21} \\ 0 & 0 & 0 & s_{22} & 1-s_{22} \\ 0 & 0 & 0 & 0 & 1 \end{bmatrix}, \mathbf{\Psi}' = \begin{bmatrix} \psi_{111} & 1-\psi_{111} & 0 & 0 & 0 \\ 0 & 0 & 1-\psi_{122} & \psi_{122} & 0 \\ \psi_{211} & 1-\psi_{211} & 0 & 0 & 0 \\ 0 & 0 & 1-\psi_{222} & \psi_{222} & 0 \\ 0 & 0 & 0 & 0 & 1 \end{bmatrix},$$

corresponding to survival and movements between sites conditional on survival respectively. Then $\mathbf{\Phi} = \mathbf{S}\mathbf{\Psi}'$. The two elementary event matrices are

$$(\mathbf{B}^0)' = \begin{bmatrix} 0 & 1 & 0 \\ 0 & 0 & 1 \\ 0 & 1 & 0 \\ 0 & 0 & 1 \\ 1 & 0 & 0 \end{bmatrix}$$

$$(\mathbf{B})' = \begin{bmatrix} 1-p_1 & p_1 & 0 \\ 1-p_2 & 0 & p_2 \\ 1-p_1 & p_1 & 0 \\ 1-p_2 & 0 & p_2 \\ 1 & 0 & 0 \end{bmatrix}$$

Here we have displayed the transpose to correspond to the matrix orientation used in E-SURGE.

To fit this model in E-SURGE we have to execute five main stages. In the first stage, we implement the structure of the model (see Figure 2) with :

1. the definition of the number of events and states;
2. the definition of the general structure of the matrices of parameters using the GEPAT interface. The different matrices are represented symbolically using an 'excel-like' interface; this step consists in filling different cells using the following rules:
   - a Greek letter indicates a parameter of interest i.e. one that will be estimated or fixed;
   - '*' indicates the complementary parameter (there is one and only one '*' by row due to the row-stochasticity of the matrices);
   - '-' indicates parameters constrained to zero.

In the second stage, we define the constraints of the model using the GEMACO interface as shown in Figure 3. Once again, particular attention must be paid to constraints applied to the event probabilities: the first encounter corresponds to $\mathbf{B}^0$ (denoted 'a(1)' or 'firste' in the GEMACO syntax) and the subsequent encounters correspond to $\mathbf{B}$ (denoted 'a(2)' or 'nexte' in the GEMACO syntax)

When the optimization of a new model stops then the program looks for parameter redundancy using the Symbolic-Numerical method (Choquet and Cole 2012). This is done by analyzing the singular values and vectors of the derivative matrix at five points in the neighborhood of the MLE

Figure 2: In window (1), we specified the number of states ($N = 5$), events ($U = 3$), age classes ($A = 1$) and groups ($G = 1$). The GEPAT interface is then opened. Patterns of $\mathbf{\Pi}$, $\mathbf{S}$, $\mathbf{\Psi}$ and $\mathbf{B}$ are defined in windows 2a, 2b, 2c, 2d, respectively using the following rules: the parameters of interest are indicated using any Greek letter, the parameters constrained to zero are indicated using '-' and the complementary parameters are indicated using '*'.

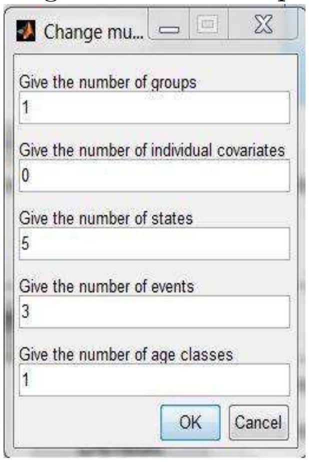

(1)

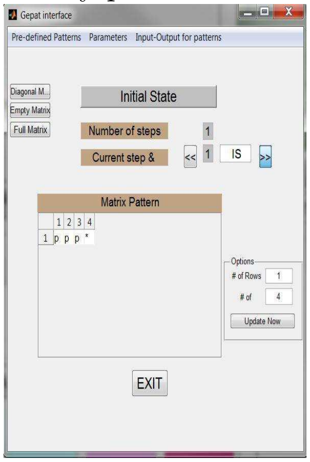

(2a)

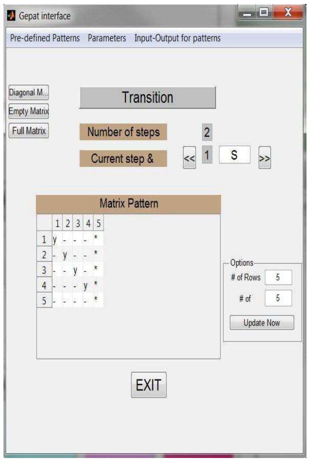

(2b)

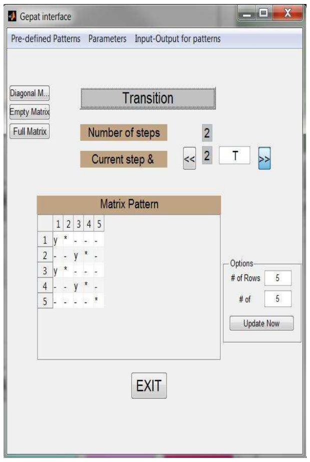

(2c)

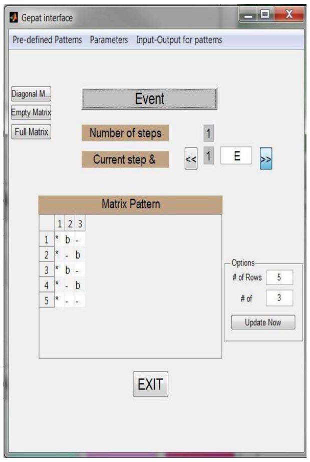

(2d)

Figure 3: The GEMACO interface defines constraints. In window (a), the user has entered the letter ‘i’ to define a constant initial state probability. In window (b), the user has entered the letter ‘i’ to define a constant survival. In window (c), the user has entered the letter ‘f’ to define a state effect for the transition. In window (d), the user has entered the phrase ‘firste+nexte.f(1 3, 2 4)’. The first mathematical parameter corresponding to ‘firste’ is fixed to 0 in the next stage.

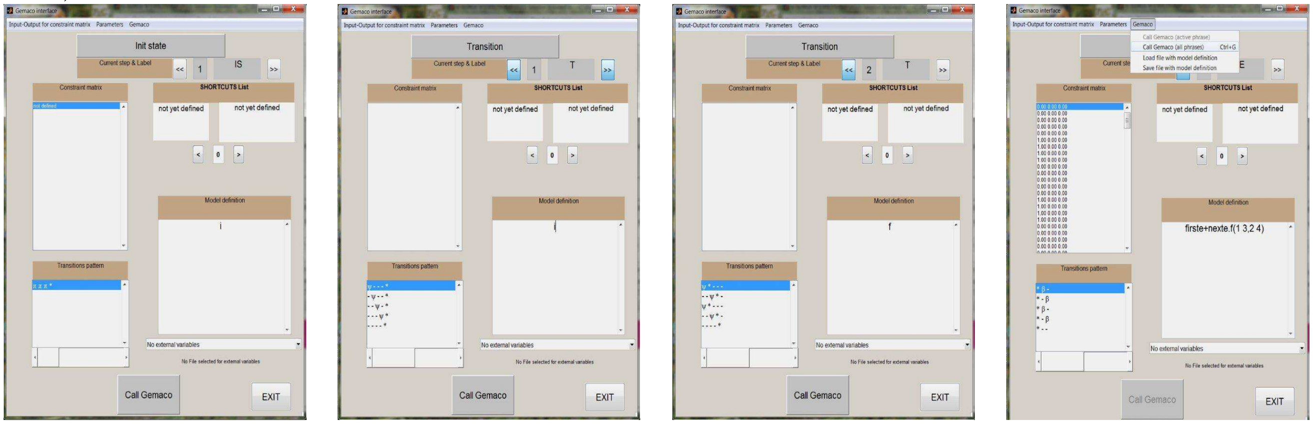

(a) (b) (c) (d)

and at the MLE itself. The estimated rank at each of the five points is shown in the DOS window. The output file also lists any parameters that may be parameter redundant.

In the next three stages, we proceed thus:

- we fix the parameter of **B** corresponding to the probability as ‘not seen’ (0) when first encountered (on the probability scale) and change the initial values if needed using the IVFV interface;
- we run the model;
- we examine and interpret the results.

7 References

Catchpole, E. A. and Morgan, B. J. T. (1997) Detecting parameter redundancy. *Biometrika*, **84**, 187-196.

Choquet, R., and Cole, D. J. (2012) A hybrid symbolic-numerical method for determining model structure. *Mathematical Biosciences*, 236, 117-125.

Choquet, R., Lebreton, J.D., Gimenez, O., Reboulet, A.M. and Pradel R. (2009). U-CARE: Utilities for performing goodness of fit tests and manipulating CApture-REcapture data. *Ecography*, **32**, 1071-1074

Cole, D. J., Morgan, B. J. T. and Titterington, D. M. (2010) Determining the parametric structure of models. *Mathematical Biosciences*, **228**, 16-30.

Cole, D. J. (2012) Determining parameter redundancy of multi-state mark-recapture models for sea birds. *Journal of Ornithology*, 152(Suppl 2), S305-S315.

Table 5: Parameter estimates and their standard error (SE) for the models chosen by score tests. M denotes the data set which has memory; $\bar{M}$ denotes the data set which has no memory.

	$\bar{M}$			M	
	Estimate	SE		Estimate	SE
π_1	0.50	0.0001	π_1	0.50	0.0001
s	0.89	0.0000	s	0.90	0.0001
ψ_{11}	0.80	0.0002	ψ_{111}	0.69	0.0012
			ψ_{122}	0.42	0.0012
ψ_{22}	0.58	0.0004	ψ_{211}	0.30	0.0007
			ψ_{222}	0.60	0.0012
p_1	0.51	0.0002	p_1	0.54	0.0009
p_2	0.32	0.0004	p_2	0.29	0.0003
