# Supplementary material for: Does your species have memory? Analyzing capture–recapture data with memory models
Source: Ecol Evol. 2014 Apr 30;4(11):2124–33. doi: 10.1002/ece3.1037 (PMC4201427; doi:10.1002/ece3.1037)
Supplement: Supplementary file 3 — Data S3. Pdf version of Maple code to for automatically creating sets of estimable parameter combinations for models AS, B and P. [file ece30004-2124-sd3.pdf]

> # Maple code for the examples paper Cole et al (Does Your Species Have Memory? Analysing Capture-Recapture Data with Memory Models.)

> # Every red arrow represents a comand line in Maple. To activate the code in Maple, press enter whilst on that line. A '#' at the start of the line means that line is just a comment.

> #To use this code, first click on the grey arrow below to open the Maple proceedures. These need activating (by pressing enter), and are called later in the code

> #Comments in green specify the main steps of the method. Blue is the Maple output. Note that a semicolon ';' indicates to Maple that results should be displayed whereas a colon ': ' indicates that results should **not** be displayed.

This section contains all the Maple procedures for finding derivative matrices and exhaustive summaries etc. Click on the arrow to open the section. (This code needs to be executed for the code in later sections to run).

Model B example. This is the example in Section 2 of the main paper.

> # To execute the code below press enter for each command.

We use the parameterisation: $\pi_i^{(t)} = \mathrm{pii}_{i,\,t},\ \phi_{i,\,j,\,k}^{(t)} = \phi_{i,\,j,\,k,\,t},\ \phi_{\mathrm{star},\,j,\,k}^{(t)} = \mathrm{phis}_{j,\,k,\,t},\ \mathrm{p}_i^{(t)} = \mathrm{p}_{i,\,t}$

>

> #Step 1: We first need to create a vector containing the probability combinations that can be used to infer results about parameter redundancy. This is known as the exhaustive summary.

#The procedure exsumB(T, N) returns the exhaustve summary for model B for a specified T years and N states.

> #Here we consider the case N=2, T=3, so we assign these values to N and T

> N := 2 : T := 3 :

> #In this model all parameters are not time dependent, which puts constraints on the parameters. The code for these constraints are below:

> $constrB := seq\big(seq\big(pii_{i,\,t} = pii_i,\ i = 1..N\big),\ t = 1..T\big),\ seq\big(seq\big(p_{i,\,t} = p_i,\ i = 1..N\big),\ t = 1..T\big),$
$seq\big(seq\big(seq\big(seq\big(\phi_{i,\,j,\,k,\,t} = \phi_{i,\,j,\,k},\ i = 1..N\big), j = 1..N\big),\ k = 1..N\big),\ t = 1..T\big),$
$seq\big(seq\big(seq\big(phis_{i,\,j,\,t} = phis_{i,\,j},\ i = 1..N\big), j = 1..N\big),\ t = 1..T\big)$:

> #Then we call the exhaustive summary, and use Maple's intrinsic proceedure, eval, to evaluate our constraint. The exhustive summary is stored in kappa

> kappa := $eval(exsumB(T, N), \{constrB\})$:

> $Transpose$(kappa[1 ..8]); $Transpose$(kappa[9 ..16]); $Transpose$(kappa[17 ..21]); #This displays the vector kappa

$[pii_1, pii_1, pii_1, pii_1\, phis_{1,\,1}\, p_1, pii_1\, phis_{1,\,2}\, p_2, pii_1\, phis_{1,\,1}\, p_1, pii_1\, phis_{1,\,2}\, p_2, (1 - pii_1)\, phis_{2,\,1}\, p_1]$

$[(1 - pii_1)\, phis_{2,\,2}\, p_2, (1 - pii_1)\, phis_{2,\,1}\, p_1, (1 - pii_1)\, phis_{2,\,2}\, p_2, \phi_{1,\,1,\,1}\, p_1, \phi_{1,\,1,\,2}\, p_2, \phi_{1,\,2,\,1}\, p_1, \phi_{1,\,2,\,2}\, p_2, \phi_{2,\,1,\,1}\, p_1]$

$$\left[\phi_{2,1,2}p_2 \;\; \phi_{2,2,1}p_1 \;\; \phi_{2,2,2}p_2 \;\; p_1 \;\; p_2\right] \quad \textbf{(2.1)}$$

> #Step 2: Next we need to specify the vector of parameters, these can be entered by hand in a vector or we can use the code below, which finds all the indeterminants of the vector kappa and puts them in a vector. (The last part of the code displays the parameters.)

```
> pars := ⟨seq(indets(kappa)[i], i = 1 ..nops(indets(kappa)))⟩ : Transpose(pars[1 ..10]);
  Transpose(pars[11 ..15]);
```

$$\left[p_1 \;\; p_2 \;\; \phi_{1,1,1} \;\; \phi_{1,1,2} \;\; \phi_{1,2,1} \;\; \phi_{1,2,2} \;\; \phi_{2,1,1} \;\; \phi_{2,1,2} \;\; \phi_{2,2,1} \;\; \phi_{2,2,2}\right]$$

$$\left[pii_1 \;\; phis_{1,1} \;\; phis_{1,2} \;\; phis_{2,1} \;\; phis_{2,2}\right] \quad \textbf{(2.2)}$$

> #Step 3: Then we need to find the derivative matrix.

#The procedure Dmat(x,y) finds the derivative matrix. x is a vector containing the exhaustive summary, y is a vector containing the parameters.

```
> D1 := Dmat(kappa, pars) :
```

> # Step 4: We then find the rank of the model, which gives the number of estimable parameters. We can also calculate the deficency as the number of parameters minus the rank.
> # In the code below r is the rank of the derivative matrix, calculated using the intrinsic Maple procedure Rank. pp is q is the number of parameters and d is the deficiency. A deficiency of d = 0 indicates the model is not parameter redundant. A deficiency of d > 0 indicates the model is paramter redundant.

```
> r := Rank(D1); pp := Dimension(pars); d := Dimension(pars) − r;
```

$$r := 15$$

$$pp := 15$$

$$d := 0 \quad \textbf{(2.3)}$$

> #This model is not parameter redundant so in theory all the parameters can be estimated.

>

Model AS example. This is the example in Section ? of the supplementary material. Left click on the arrow to view this example.

> # To execute the code below press enter for each command.
> # We use the parameterisation: $\pi_i^{(t)} = pii_{i,t}$, $\phi_{i,j}^{(t)} = \phi_{i,j,t}$, $p_i^{(t)} = p_{i,t}$

> #Step 1: We first need to create a vector containing the probability combinations that can be used to infer results about parameter redundancy. This is known as the exhaustive summary.
> #The procedure exsumAS(T, N) returns the exhaustive summary for model AS for the specified T years and N states.

> #Here we consider the case N=2, T=3, so we assign these values to N and T

```
> N := 2 : T := 3 :
```

> #In this model all p are are not time dependent, which puts a constraint on the parameters. The code for this constraint is below:

```
> constrA := seq(seq(p[i, t] = p[i], i = 1 ..N), t = 1 ..T) :
```

> #Then we call the exhaustive summary, and use Maple's intrinsic proceedure, eval, to evaluate our constraint. The exhustive summary is stored in kappa

> kappa := *eval*(*exsumAS*(*T*, *N*), {constrA}) :

> *Transpose*(kappa[1 ..8]); *Transpose*(kappa[9 ..13]); #This displays the vector kappa

$$\left[pii_{1,1}, pii_{1,2}, pii_{1,3}, pii_{1,1}\,\phi_{1,1,1}\,p_1, pii_{1,1}\,\phi_{1,2,1}\,p_2, pii_{1,2}\,\phi_{1,1,2}\,p_1, pii_{1,2}\,\phi_{1,2,2}\,p_2, \left(1 - pii_{1,1}\right)\phi_{2,1,1}\,p_1\right]$$

$$\left[\begin{array}{ccccc} \left(1-pii_{1,1}\right)\phi_{2,2,1}\,p_2 & \left(1-pii_{1,2}\right)\phi_{2,1,2}\,p_1 & \left(1-pii_{1,2}\right)\phi_{2,2,2}\,p_2 & p_1 & p_2 \end{array}\right] \quad \textbf{(3.1)}$$

> #Step 2: Next, we need to specify the vector of parameters. These can be entered by hand in a vector or we can use the code below, which finds all the indeterminants of the vector kappa and puts them in a vector. (The last part of the code displays the parameters.)

> *pars* := ⟨*seq*(*indets*(kappa)[*i*], *i* = 1 ..*nops*(*indets*(kappa)))⟩ :

> *Transpose*(pars[1 ..8]); *Transpose*(pars[9 ..13]); #This displays the vector of parameters

$$\left[\begin{array}{cccccccc} p_1 & p_2 & \phi_{1,1,1} & \phi_{1,1,2} & \phi_{1,2,1} & \phi_{1,2,2} & \phi_{2,1,1} & \phi_{2,1,2} \end{array}\right]$$

$$\left[\begin{array}{ccccc} \phi_{2,2,1} & \phi_{2,2,2} & pii_{1,1} & pii_{1,2} & pii_{1,3} \end{array}\right] \quad \textbf{(3.2)}$$

> #Step 3: Then we need to find the derivative matrix.
>
> #The procedure Dmat(x,y) finds the derivative matrix. x is a vector containing the exhaustive summary, y is a vector containing the parameters.

> *D1* := *Dmat*(kappa, *pars*) :

> # Step 4: We then find the rank of the model, which gives the number of estimable parameters. We can also calculate the deficency as the number of parameters minus the rank.
> # In the code below r is the rank of the derivative matrix, calculated using the intrinsic Maple procedure Rank. pp is q is the number of parameters and d is the deficiency. A deficiency of d = 0 indicates the model is not parameter redundant. A deficiency of d > 0 indicates the model is paramter redundant.

> *r* := *Rank*(*D1*); *pp* := *Dimension*(*pars*); *d* := *Dimension*(*pars*) − *r*;

$$r := 13$$
$$pp := 13$$
$$d := 0 \quad \textbf{(3.3)}$$

> #This model is not parameter redundant so in theory all the parameters can be estimated.

> #Step 5: Next we consider generalising this result to any number of years. We need to increase T by 1 so that we have:

> *T* := 4 : *N* := 2 :

> *constrA* := *seq*(*seq*($p_{i,t} = p_i$, *i* = 1 ..*N*), *t* = 1 ..*T*) : #The same constraints as before

> #Step 6: Increasing T adds new extra terms to our exhaustive summary. We need to create a vector of the additional terms under the exhaustive summary. This is stored in the vector kappaex

> *kappa* := *eval*(*exsumAS*(*T*, *N*), {*constrA*}) :

> *kappaex* := ⟨kappa[4], kappa[9], kappa[10], kappa[15], kappa[16]⟩;

$$kappaex := \begin{bmatrix} pii_{1,4} \\ pii_{1,3}\,\phi_{1,1,3}\,p_1 \\ pii_{1,3}\,\phi_{1,2,3}\,p_2 \\ (1-pii_{1,3})\,\phi_{2,1,3}\,p_1 \\ (1-pii_{1,3})\,\phi_{2,2,3}\,p_2 \end{bmatrix} \quad \textbf{(3.4)}$$

> #Step 7: We need to create a vector of the additional terms parameters. This is stored in the vector parex

> $parex := \langle pii_{1,4}, \phi_{1,1,3}, \phi_{1,2,3}, \phi_{2,1,3}, \phi_{2,2,3} \rangle :$

> #Step 8: Then we need to form the derivative matrix from just the extra exhaustive summary terms and extra parameters

> $Dex := Dmat(kappaex, parex);$ # The extra derivative matrix

$$Dex := \begin{bmatrix} 1 & 0 & 0 & 0 & 0 \\ 0 & pii_{1,3}\,p_1 & 0 & 0 & 0 \\ 0 & 0 & pii_{1,3}\,p_2 & 0 & 0 \\ 0 & 0 & 0 & (1-pii_{1,3})\,p_1 & 0 \\ 0 & 0 & 0 & 0 & (1-pii_{1,3})\,p_2 \end{bmatrix} \quad \textbf{(3.5)}$$

> #Step 9: We need to check that Dex is full rank, ie the deficiency is 0:

> $r := Rank(Dex); pp := Dimension(parex); d := Dimension(parex) - r;$

$$r := 5$$
$$pp := 5$$
$$d := 0 \quad \textbf{(3.6)}$$

> # As the extra derivative matrix is full rank too by the extension theorem the model for any T will be full rank, ie the deficiency is always 0.

> #We can follow identical steps for increasing the number of sites. We increase N to 3.

> $T := 3 : N := 3 :$

> $constrA := seq\big(seq\big(p_{i,t} = p_i, i = 1..N\big), t = 1..T\big) :$

> kappa $:= eval(exsumAS(T, N), \{constrA\}) :$

> $kappaex :=$ ⟨kappa[2], kappa[4], kappa[6], kappa[9], kappa[12], kappa[15], kappa[18], kappa[19], kappa[20], kappa[21], kappa[22], kappa[23], kappa[24], kappa[27]⟩ :

> $parex := \langle p_3, \phi_{1,3,1}, \phi_{1,3,2}, \phi_{2,3,1}, \phi_{2,3,2}, \phi_{3,1,1}, \phi_{3,1,2}, \phi_{3,2,1}, \phi_{3,2,2}, \phi_{3,3,1}, \phi_{3,3,2}, pii_{2,2}, pii_{2,3} \rangle :$

> $Dex := Dmat(kappaex, parex) :$

> $r := Rank(Dex); pp := Dimension(parex); d := Dimension(parex) - r;$

$$r := 13$$
$$pp := 13$$
$$d := 0 \quad \textbf{(3.7)}$$

> # As the extra derivative matrix is full rank too by the extension theorem the model for any N will be full rank, ie the deficiency is always 0.

>

Model P example. This is the example in Section ? of the supplementary material. Left click on the arrow to view this example.

> # To execute the code below press enter for each command.
We use the parameterisation: $\pi^{(t)}_{i,j} = \mathrm{pii}_{i,j,t}$, $\phi^{(t)}_{i,j,k} = \phi_{i,j,k,t}$, $p^{(t)}_i = p_{i,t}$

> #Step 1: We first need to create a vector containing the probability combinations that can be used to infer results about parameter redundancy. This is known as the exhaustive summary.
#The procedure exsumP(T, N) returns the exhaustve summary for model P for a specified T years and N states.

> #Here we consider the case N=2, T=3, so we assign these values to N and T

```
> N := 2 : T := 3 :
```

> #In this model p_i and $\pi_{i,j}$ are not dependent on time. The code for this constraint is below :

$$constrP := seq\left(seq\left(p_{i,t}=p_i, i=1..N\right), t=1..T\right), seq\left(seq\left(seq\left(pii_{i,j,t}=pii_{i,j}, t=1..T\right), i=1..N\right), j=1..N\right) :$$

> #Then we call the exhaustive summary, and use Maple's intrinsic proceedure, eval, to evaluate our constraint. The exhustive summary is stored in kappa

$$\text{kappa} := eval(exsumP(T, N), \{constrP\}) :$$

> $Transpose(\text{kappa}[1..8])$; $Transpose(\text{kappa}[9..17])$; $Transpose(\text{kappa}[17..19])$; #This displays the vector kappa

$$[pii_{1,1}+pii_{2,1}, pii_{1,1}+pii_{2,1}, pii_{1,1}+pii_{2,1}, pii_{2,1}, pii_{1,2}, pii_{1,1}\,\phi_{1,1,1,1}\,p_1 + pii_{2,1}\,\phi_{2,1,1,1}\,p_1, pii_{1,1}\,\phi_{1,1,2,1}\,p_2 + pii_{2,1}\,\phi_{2,1,2,1}\,p_2, pii_{1,2}\,\phi_{1,2,1,1}\,p_1 + (1-pii_{1,1}-pii_{1,2}-pii_{2,1})\,\phi_{2,2,1,1}\,p_1]$$

$$[pii_{1,2}\,\phi_{1,2,2,1}\,p_2 + (1-pii_{1,1}-pii_{1,2}-pii_{2,1})\,\phi_{2,2,2,1}\,p_2, \phi_{1,1,1,2}\,p_1, \phi_{1,1,2,2}\,p_2, \phi_{1,2,1,2}\,p_1, \phi_{1,2,2,2}\,p_2, \phi_{2,1,1,2}\,p_1, \phi_{2,1,2,2}\,p_2, \phi_{2,2,1,2}\,p_1, \phi_{2,2,2,2}\,p_2]$$

$$\left[\begin{array}{ccc} \phi_{2,2,2,2}\,p_2 & p_1 & p_2 \end{array}\right] \quad \mathbf{(4.1)}$$

> #Step 2: Next, we need to specify the vector of parameters. These can be entered by hand in a vector or we can use the code below, which finds all the indeterminants of the vector kappa and puts them in a vector. (The last part of the code displays the parameters.)

> $pars := \langle seq(indets(\text{kappa})[i], i=1..nops(indets(\text{kappa})))\rangle : Transpose(pars[1..10])$; $Transpose(pars[11..20])$; $Transpose(pars[21..21])$;
#Find parameters and displays them

$$\left[\begin{array}{cccccccccc} p_1 & p_2 & \phi_{1,1,1,1} & \phi_{1,1,1,2} & \phi_{1,1,2,1} & \phi_{1,1,2,2} & \phi_{1,2,1,1} & \phi_{1,2,1,2} & \phi_{1,2,2,1} & \phi_{1,2,2,2} \end{array}\right]$$

$$\left[\begin{array}{cccccccccc} \phi_{2,1,1,1} & \phi_{2,1,1,2} & \phi_{2,1,2,1} & \phi_{2,1,2,2} & \phi_{2,2,1,1} & \phi_{2,2,1,2} & \phi_{2,2,2,1} & \phi_{2,2,2,2} & pii_{1,1} & pii_{1,2} \end{array}\right]$$

$$\left[\, pii_{2,1} \,\right] \quad (4.2)$$

> #Step 3: Then we need to find the derivative matrix.

#The proceedure Dmat(x,y) finds the derivative matrix. x is a vector containing the exhaustive summary, y is a vector containing the parameters.

> $D1 := Dmat(\text{kappa}, pars)$:

> # In the code below r is the rank of the derivative matrix, calculated using the internal Maple proceedure Rank. pp is q is the number of parameters and d is the deficency. A deficency of d = 0 indicates the model is not parameter redundant. A deficency of d > 0 indicates the model is paramter redundant.

> # Step 4: We then find the rank of the model, which gives the number of estimable parameters. We can also calculate the deficency as the number of parameters minus the rank.
In the code below r is the rank of the derivative matrix, calculated using the intrinsic Maple procedure Rank. pp is q is the number of parameters and d is the deficiency. A deficiency of d = 0 indicates the model is not parameter redundant. A deficiency of d > 0 indicates the model is paramter redundant.

> $r := Rank(D1); pp := Dimension(pars); d := Dimension(pars) - r;$

$$r := 17$$

$$pp := 21$$

$$d := 4 \quad (4.3)$$

> #This model is parameter redundant with deficiency 4

> #Step 5: In a parameter redundant model we can find the estimable parameter combinations using the proceedure Estpars(D1, pars), for a derivative matrix D1 and parameter vectors pars

> $Estpars(D1, pars)$

$$\Big\{ f\big(p_1, p_2, \phi_{1,1,1,1}, \phi_{1,1,1,2}, \phi_{1,1,2,1}, \phi_{1,1,2,2}, \phi_{1,2,1,1}, \phi_{1,2,1,2}, \phi_{1,2,2,1}, \phi_{1,2,2,2}, \phi_{2,1,1,1}, \phi_{2,1,1,2}, \phi_{2,1,2,1}, \phi_{2,1,2,2}, \phi_{2,2,1,1}, \phi_{2,2,1,2}, \phi_{2,2,2,1}, \phi_{2,2,2,2}, pii_{1,1}, pii_{1,2}, pii_{2,1}\big) = \_F1\Big(p_1, p_2, \phi_{1,1,1,2}, \phi_{1,1,2,2}, \phi_{1,2,1,2}, \phi_{1,2,2,2}, \phi_{2,1,1,2}, \phi_{2,1,2,2}, \phi_{2,2,1,2}, \phi_{2,2,2,2}, pii_{1,1}, pii_{1,2}, pii_{2,1}, \frac{(-1 + pii_{1,1} + pii_{1,2} + pii_{2,1})\,\phi_{2,2,2,1} - pii_{1,2}\,\phi_{1,2,2,1}}{-1 + pii_{1,1} + pii_{1,2} + pii_{2,1}}, \frac{(-1 + pii_{1,1} + pii_{1,2} + pii_{2,1})\,\phi_{2,2,1,1} - pii_{1,2}\,\phi_{1,2,1,1}}{-1 + pii_{1,1} + pii_{1,2} + pii_{2,1}}, \frac{pii_{1,1}\,\phi_{1,1,2,1} + pii_{2,1}\,\phi_{2,1,2,1}}{pii_{2,1}}, \frac{pii_{1,1}\,\phi_{1,1,1,1} + pii_{2,1}\,\phi_{2,1,1,1}}{pii_{2,1}}\Big)\Big\} \quad (4.4)$$

> #Step 6: To generalise results for parameter redundant models we first need to reparameterise using the estimable parameter combinations.

#We set $g_1 = \frac{(-1+pii_{1,1}+pii_{1,2}+pii_{2,1})\,\phi_{2,2,1,1} - pii_{1,2}\,\phi_{1,2,1,1}}{-1+pii_{1,1}+pii_{1,2}+pii_{2,1}}$ etc...

> #The reparameterised model exhaustive summary is found using:

> $kappar := simplify\left(eval\left(\text{kappa}, \left\{\phi_{1,1,1,1} = \frac{pii_{2,1}\,(g_4-\phi_{2,1,1,1})}{pii_{1,1}}, \phi_{1,1,2,1} = \frac{pii_{2,1}\,(g_3-\phi_{2,1,2,1})}{pii_{1,1}}, \phi_{2,2,1,1} = \frac{pii_{1,2}\,\phi_{1,2,1,1} - g_2 + g_2\,pii_{1,1} + g_2\,pii_{1,2} + g_2\,pii_{2,1}}{-1+pii_{1,1}+pii_{1,2}+pii_{2,1}}, \phi_{2,2,2,1} = \frac{pii_{1,2}\,\phi_{1,2,2,1} - g_1 + g_1\,pii_{1,1} + g_1\,pii_{1,2} + g_1\,pii_{2,1}}{-1+pii_{1,1}+pii_{1,2}+pii_{2,1}}\right\}\right)\right):$

> #The new set of parameters are:

> $parsre := \langle seq(indets(kappar)[i], i=1..nops(indets(kappar)))\rangle : Transpose(parsre[1..10]); Transpose(parsre[11..17]);$#Find parameters and displays them

$$\left[\begin{array}{cccccccccc} g_1 & g_2 & g_3 & g_4 & p_1 & p_2 & \phi_{1,1,1,2} & \phi_{1,1,2,2} & \phi_{1,2,1,2} & \phi_{1,2,2,2}\end{array}\right]$$

$$\left[\begin{array}{ccccccc} \phi_{2,1,1,2} & \phi_{2,1,2,2} & \phi_{2,2,1,2} & \phi_{2,2,2,2} & pii_{1,1} & pii_{1,2} & pii_{2,1}\end{array}\right] \qquad \textbf{(4.5)}$$

> #The derivative matrix should be full rank (have deficiency 0) and this is confirmed below:

> $Dr := Dmat(kappar, parsre):$

> $r := Rank(Dr); q := Dimension(parsre); d := Dimension(parsre) - r;$

$$r := 17$$
$$q := 17$$
$$d := 0 \qquad \textbf{(4.6)}$$

> #Step 7: Next we can generalise the reparameterised model. Adding an extra year of data gives:

> $\text{N} := 2 : \text{T} := 4 :$

> $constrP := seq\left(seq\left(p_{i,t} = p_i, i=1..N\right), t=1..T\right), seq\left(seq\left(seq\left(pii_{i,j,t} = pii_{i,j}, t=1..T\right), i=1..N\right), j=1..N\right):$

> $\text{kappa} := eval(exsumP(T, N), \{constrP\}):$

> $kappar := simplify\left(eval\left(\text{kappa}, \left\{\phi_{1,1,1,1} = \frac{pii_{2,1}\,(g_4-\phi_{2,1,1,1})}{pii_{1,1}}, \phi_{1,1,2,1} = \frac{pii_{2,1}\,(g_3-\phi_{2,1,2,1})}{pii_{1,1}}, \phi_{2,2,1,1} = \frac{pii_{1,2}\,\phi_{1,2,1,1} - g_2 + g_2\,pii_{1,1} + g_2\,pii_{1,2} + g_2\,pii_{2,1}}{-1+pii_{1,1}+pii_{1,2}+pii_{2,1}}, \phi_{2,2,2,1} = \frac{pii_{1,2}\,\phi_{1,2,2,1} - g_1 + g_1\,pii_{1,1} + g_1\,pii_{1,2} + g_1\,pii_{2,1}}{-1+pii_{1,1}+pii_{1,2}+pii_{2,1}}\right\}\right)\right):$

> $kapparex := \langle kappar[7], kappar[21], kappar[22], kappar[23], kappar[24], kappar[25],$

$kappar[26], kappar[27], kappar[28]\rangle$:

> $parex := \langle \phi_{1,1,1,3}, \phi_{1,1,2,3}, \phi_{1,2,1,3}, \phi_{1,2,2,3}, \phi_{2,1,1,3}, \phi_{2,1,2,3}, \phi_{2,2,1,3}, \phi_{2,2,2,3}, pii_{1,2}\rangle$:

> $Drex := Dmat(kapparex, parex)$:

> $r := Rank(Drex); q := Dimension(parex); d := Dimension(parex) - r;$

$$r := 9$$

$$q := 9$$

$$d := 0 \quad \textbf{(4.7)}$$

> #As the extended part is full rank, by the extension theorem the reparameterised model will always be full have rank 8T−7. By the reparameterisation Theorem the origional parameterised model will also have rank 8T−7, but there are 8T−3 parameters, so the deficiency will always be 4.

>
