# Supplementary material for: Does your species have memory? Analyzing capture–recapture data with memory models
Source: Ecol Evol. 2014 Apr 30;4(11):2124–33. doi: 10.1002/ece3.1037 (PMC4201427; doi:10.1002/ece3.1037)
Supplement: Supplementary file 5 — Data S5. Pdf version of Maple code examples. [file ece30004-2124-sd5.pdf]

```
> #This is Maple code for the paper Does Your Species Have Memory? Analysing Capture-
      Recapture Data with Memory Models., by Cole et al,
  #This code can be used to check whether a particular data set is parameter redundant or not
  #First press enter to activate all of the inital procedures then follow the examples below
> restart;
> with(LinearAlgebra) :
> #Below are proceedures used in the worksheet below (NB need to be activated by pressing enter)
> Dmat :=proc(se, pars)
  local DD1, i, j;
  description "Form the derivative matrix";
  with(LinearAlgebra) :
  DD1 := Matrix(1 ..Dimension(pars), 1 ..Dimension(se)) :
  for i from 1 to Dimension(pars)  do
      for j from 1 to Dimension(se) do
          DD1[i, j] := diff(se[j], pars[i])
      end do
   end do;
  DD1;
  end proc:
> Findkappa :=proc(H, Pit, Phi0, Phit, B0, B, T)
   local j, e, kappa, h, indi, k, Ph, i;

      description "Given the matrices and a matrix of unique histories this procedure returns the
      exhaustive summary";
  κ := Vector(Dimension(H)[1]) :
  for j from 1 to Dimension(H)[1] do
      h := Row(H, j) :
      # e is first nonzero entry of h
      indi := 0 :
      for k from 1 to Dimension(H)[2] do
          if indi = 0 then
             if h[k] ≠ 0 then
                e := k :
                indi := 1 :
             end if:
          end if:
      end do:
         Ph := Multiply(eval(Pit, t = e), DiagonalMatrix(Row(B0, h[e] + 1)));
      for i from e + 1 to T do
          if i − 1 = e then
             Ph := Multiply(Ph, Multiply(eval(Phit0, t = i − 1), DiagonalMatrix(Row(eval(B, t
       = i), h[i] + 1)))) :
          else
             Ph := Multiply(Ph, Multiply(eval(Phit, t = i − 1), DiagonalMatrix(Row(eval(B, t = i),
      h[i] + 1)))) :
          end if:
      end do:
      Ph := Multiply(Ph, Vector(Dimension(Ph), 1)) :
      κ[j] := Ph :
   end do:
```

```
kappa;
end proc:
> Hybrid :=proc(D1, pars)
local results, j, numpars, D1rand :

    description   "This procedure finds the rank and deficency using the hybrid-symbolic-
    numeric method";
results := Matrix(5, 1) :
for j from 1 to 5 do
   numpars := seq( pars[i] = evalf( rand( ) / 1000000000000 ), i = 1 ..Dimension(pars) ) :
   D1rand := eval(D1, {numpars});
   results[j, 1] := Rank( D1rand);
   end do:
 <max(results)|Dimension(pars) - max(results)> :
 end proc:
>
> #This example is for 2 sites using model AS
> #First enter all of the probability matrices,  including any constraints, as per table 2 of the paper:
```

> #Here we use Pit for Π_t and pii[i, t] for $\pi_{i,t}$ and this is time dependent. We use Phit and Phit0 for Φ_t and Φ_t^0 and these are constant. p_i is also constant.

```
> Pit := <pii[1, t]|1 - pii[1, t]|0>;
```

$$Pit := \begin{bmatrix} pii_{1,t} & 1-pii_{1,t} & 0 \end{bmatrix} \quad (1)$$

```
> Phit := <<phi[1, 1]|phi[1, 2]|1 - phi[1, 1] - phi[1, 2]>, <phi[2, 1]|phi[2, 2]|1 - phi[2, 1]
    - phi[2, 2]>, <0|0|1>>;
```

$$Phit := \begin{bmatrix} \phi_{1,1} & \phi_{1,2} & 1-\phi_{1,1}-\phi_{1,2} \\ \phi_{2,1} & \phi_{2,2} & 1-\phi_{2,1}-\phi_{2,2} \\ 0 & 0 & 1 \end{bmatrix} \quad (2)$$

```
> Phit0 := Phit:
> B0 := <<0|0|1>, <1|0|0>, <0|1|0>>;
```

$$B0 := \begin{bmatrix} 0 & 0 & 1 \\ 1 & 0 & 0 \\ 0 & 1 & 0 \end{bmatrix} \quad (3)$$

```
> B := <<1 - p[1, t]|1 - p[2, t]|1>, <p[1, t]|0|0>, <0|p[2, t]|0>> ;
```

$$B := \begin{bmatrix} 1-p_{1,t} & 1-p_{2,t} & 1 \\ p_{1,t} & 0 & 0 \\ 0 & p_{2,t} & 0 \end{bmatrix} \quad (4)$$

```
> #Then specify how many year of data and the number of sites, in this case there are T=5 years of
    data and N=2 sites.
> T := 5 : N := 2 :
```

> #Then input the unique histories for your data set. (The Mark format for this data set is given in simplesimexample.inp. The easiest way to do this from an .inp file is to open that file in excel and specify that there is a new column after every 0, 1 or 2, then copy and paste the data from excel, there is no need to input the [ ] brackets.)

$$H := \begin{bmatrix}
0 & 0 & 0 & 0 & 1 \\
0 & 0 & 0 & 0 & 2 \\
0 & 0 & 0 & 1 & 0 \\
0 & 0 & 0 & 1 & 1 \\
0 & 0 & 0 & 1 & 2 \\
0 & 0 & 0 & 2 & 0 \\
0 & 0 & 0 & 2 & 1 \\
0 & 0 & 0 & 2 & 2 \\
0 & 0 & 1 & 0 & 0 \\
0 & 0 & 1 & 0 & 1 \\
0 & 0 & 1 & 0 & 2 \\
0 & 0 & 1 & 1 & 0 \\
0 & 0 & 1 & 1 & 2 \\
0 & 0 & 1 & 2 & 0 \\
0 & 0 & 1 & 2 & 1 \\
0 & 0 & 1 & 2 & 2 \\
0 & 0 & 2 & 1 & 1 \\
0 & 0 & 2 & 2 & 0 \\
0 & 1 & 0 & 0 & 0 \\
0 & 1 & 0 & 0 & 2 \\
0 & 1 & 0 & 1 & 0 \\
0 & 1 & 1 & 0 & 0 \\
0 & 1 & 1 & 0 & 1 \\
0 & 1 & 1 & 1 & 0 \\
0 & 1 & 1 & 1 & 2 \\
0 & 1 & 2 & 1 & 0 \\
0 & 2 & 0 & 0 & 0 \\
0 & 2 & 0 & 0 & 1 \\
0 & 2 & 0 & 0 & 2 \\
0 & 2 & 0 & 1 & 0 \\
0 & 2 & 0 & 1 & 1 \\
0 & 2 & 0 & 2 & 2 \\
0 & 2 & 1 & 0 & 1 \\
0 & 2 & 1 & 1 & 0
\end{bmatrix}:$$

```
> #We find the probabiltiies of each history using the code:
> kappa := Findkappa(H, Pit, Phi0, Phit, B0, B, T) :
> #Then we can find which parameters are present in the model using the following code:
> pars := ⟨seq(indets(kappa)[i], i = 1 ..nops(indets(kappa)))⟩ :
> #The derivative matrix is found using the following code:
> D1 := Dmat(kappa, pars) :
> #We find the rank and deficency using the hybrid method using the following code (the first
    number is the rank the second is the deficency):
> Hybrid(D1, pars);
```

$$\begin{bmatrix} 17 & 0 \end{bmatrix} \tag{5}$$

```
> ##The deficency is still 0 (not parameter redundant) for this data set
>
> #This example is for 2 sites using model B
> #First enter all of the probability matrices,  including any constraints, as per table 2 of the paper:
```

> #Here we use Pit for Π_t and pii[i] for π_i and this is constant. We use Phit and Phit0 for Φ_t and Φ_t^0 and phis[i, j, t] for $\phi^{(t)}_{*, i, j}$ and these are time dependent. p_i is also constant.

```
> Pit := ⟨pii[1]|1 − pii[1]|0⟩;
```

$$Pit := \begin{bmatrix} pii_1 & 1 - pii_1 & 0 \end{bmatrix} \tag{6}$$

```
> Phit0 := ⟨⟨phis[1, 1, t]|phis[1, 2, t]|0|0| 1 − phis[1, 1, t] − phis[1, 2, t]⟩, ⟨0|0 |phis[2, 1, t]
    |phis[2, 2, t]|1 − phis[2, 1 , t] − phis[2, 2, t]⟩, ⟨0|0|0|0|1⟩⟩;
```

$$Phit0 := \begin{bmatrix} phis_{1,1,t} & phis_{1,2,t} & 0 & 0 & 1 - phis_{1,1,t} - phis_{1,2,t} \\ 0 & 0 & phis_{2,1,t} & phis_{2,2,t} & 1 - phis_{2,1,t} - phis_{2,2,t} \\ 0 & 0 & 0 & 0 & 1 \end{bmatrix} \tag{7}$$

```
> Phit := ⟨⟨phi[1, 1, 1, t]|phi[1, 1, 2, t]|0|0|1 − phi[1, 1, 1, t] − phi[1, 1, 2, t]⟩, ⟨ 0|0|phi[1, 2, 1,
    t]|phi[1, 2, 2, t]|1 − phi[1, 2, 1 , t] − phi[1, 2, 2, t]⟩, ⟨phi[2, 1, 1, t]|phi[2, 1, 2, t]|0|0|1
    − phi[2, 1, 1, t] − phi[2, 1, 2, t]⟩, ⟨ 0|0|phi[2, 2, 1, t]|phi[2, 2, 2, t]|1 − phi[2, 2, 1 , t]
    − phi[2, 2, 2, t]⟩, ⟨0|0|0|0|1⟩⟩;
```

$$Phit := \begin{bmatrix} \phi_{1,1,1,t} & \phi_{1,1,2,t} & 0 & 0 & 1 - \phi_{1,1,1,t} - \phi_{1,1,2,t} \\ 0 & 0 & \phi_{1,2,1,t} & \phi_{1,2,2,t} & 1 - \phi_{1,2,1,t} - \phi_{1,2,2,t} \\ \phi_{2,1,1,t} & \phi_{2,1,2,t} & 0 & 0 & 1 - \phi_{2,1,1,t} - \phi_{2,1,2,t} \\ 0 & 0 & \phi_{2,2,1,t} & \phi_{2,2,2,t} & 1 - \phi_{2,2,1,t} - \phi_{2,2,2,t} \\ 0 & 0 & 0 & 0 & 1 \end{bmatrix} \tag{8}$$

```
> B0 := ⟨⟨0|0|1⟩, ⟨1|0|0⟩, ⟨0|1|0⟩⟩;
```

$$B0 := \begin{bmatrix} 0 & 0 & 1 \\ 1 & 0 & 0 \\ 0 & 1 & 0 \end{bmatrix} \tag{9}$$

> $B := \langle\langle 1 - p[1] | 1 - p[2] | 1 - p[1] | 1 - p[2] | 1\rangle, \langle p[1] | 0 | p[1] | 0 | 0\rangle, \langle 0 | p[2] | 0 | p[2] | 0\rangle\rangle$;

$$B := \begin{bmatrix} 1-p_1 & 1-p_2 & 1-p_1 & 1-p_2 & 1 \\ p_1 & 0 & p_1 & 0 & 0 \\ 0 & p_2 & 0 & p_2 & 0 \end{bmatrix} \quad \textbf{(10)}$$

>

> #Then specify how many year of data and the number of sites, in this case there are T=5 years of data and N=2 sites.

> T := 5 : N := 2 :

> #Then input the unique histories for your data set. (The Mark format for this data set is given in simplesimexample.inp. The easiest way to do this from an .inp file is to open that file in excel and specify that there is a new column after every 0, 1 or 2, then copy and paste the data from excel, there is no need to input the [ ] brackets.)

$$
> H := \begin{bmatrix}
0 & 0 & 0 & 0 & 1 \\
0 & 0 & 0 & 0 & 2 \\
0 & 0 & 0 & 1 & 0 \\
0 & 0 & 0 & 1 & 1 \\
0 & 0 & 0 & 1 & 2 \\
0 & 0 & 0 & 2 & 0 \\
0 & 0 & 0 & 2 & 1 \\
0 & 0 & 0 & 2 & 2 \\
0 & 0 & 1 & 0 & 0 \\
0 & 0 & 1 & 0 & 1 \\
0 & 0 & 1 & 0 & 2 \\
0 & 0 & 1 & 1 & 0 \\
0 & 0 & 1 & 1 & 2 \\
0 & 0 & 1 & 2 & 0 \\
0 & 0 & 1 & 2 & 1 \\
0 & 0 & 1 & 2 & 2 \\
0 & 0 & 2 & 1 & 1 \\
0 & 0 & 2 & 2 & 0 \\
0 & 1 & 0 & 0 & 0 \\
0 & 1 & 0 & 0 & 2 \\
0 & 1 & 0 & 1 & 0 \\
0 & 1 & 1 & 0 & 0 \\
0 & 1 & 1 & 0 & 1 \\
0 & 1 & 1 & 1 & 0 \\
0 & 1 & 1 & 1 & 2 \\
0 & 1 & 2 & 1 & 0 \\
0 & 2 & 0 & 0 & 0 \\
0 & 2 & 0 & 0 & 1 \\
0 & 2 & 0 & 0 & 2 \\
0 & 2 & 0 & 1 & 0 \\
0 & 2 & 0 & 1 & 1 \\
0 & 2 & 0 & 2 & 2 \\
0 & 2 & 1 & 0 & 1 \\
0 & 2 & 1 & 1 & 0
\end{bmatrix} :
$$

> #We find the probabiltiies of each history using the code:

> kappa := *Findkappa*(*H*, *Pit*, *Phi0*, *Phit*, *B0*, *B*, *T*) :

> #Then we can find which parameters are present in the model using the following code:

> *pars* := ⟨*seq*(*indets*(kappa)[*i*], *i* = 1 ..*nops*(*indets*(kappa)))⟩ :

> #The derivative matrix is found using the following code:

> *D1* := *Dmat*(kappa, *pars*) :

> #We find the rank and deficency using the hybrid method using the following code (the first number is the rank the second is the deficency):

> *Hybrid*(*D1*, *pars*);

$$\begin{bmatrix} 43 & 0 \end{bmatrix} \tag{11}$$

> #The deficency is still 0 (not parameter redundant) for this data set

>

> #This example is for 2 sites using model P

> #First enter all of the probability matrices, including any constraints, as per table 2 of the paper:

> #Here we use Pit for Π_t and pii[i] for π_i and this is constant. We use Phit and Phit0 for Φ_t and Φ_t^0 and these are time dependent. p_i is also constant.

> *Pit* := ⟨*pii*[1, 1, *t*]|*pii*[1, 2, *t*]|*pii*[2, 1, *t*]|1 − *pii*[1, 1, *t*] − *pii*[1, 2, *t*] − *pii*[2, 1, *t*]|0⟩;

$$Pit := \begin{bmatrix} pii_{1,1,t} & pii_{1,2,t} & pii_{2,1,t} & 1 - pii_{1,1,t} - pii_{1,2,t} - pii_{2,1,t} & 0 \end{bmatrix} \tag{12}$$

> *Phit* := ⟨⟨phi[1, 1, 1, *t*]|phi[1, 1, 2, *t*]|0|0|1 − phi[1, 1, 1, *t*] − phi[1, 1, 2, *t*]⟩, ⟨ 0|0|phi[1, 2, 1, *t*]|phi[1, 2, 2, *t*]|1 − phi[1, 2, 1 , *t*] − phi[1, 2, 2, *t*]⟩, ⟨phi[2, 1, 1, *t*]|phi[2, 1, 2, *t*]|0|0|1 − phi[2, 1, 1, *t*] − phi[2, 1, 2, *t*]⟩, ⟨ 0|0|phi[2, 2, 1, *t*]|phi[2, 2, 2, *t*]|1 − phi[2, 2, 1 , *t*] − phi[2, 2, 2, *t*]⟩, ⟨0|0|0|0|1⟩⟩;;

$$Phit := \begin{bmatrix} \phi_{1,1,1,t} & \phi_{1,1,2,t} & 0 & 0 & 1 - \phi_{1,1,1,t} - \phi_{1,1,2,t} \\ 0 & 0 & \phi_{1,2,1,t} & \phi_{1,2,2,t} & 1 - \phi_{1,2,1,t} - \phi_{1,2,2,t} \\ \phi_{2,1,1,t} & \phi_{2,1,2,t} & 0 & 0 & 1 - \phi_{2,1,1,t} - \phi_{2,1,2,t} \\ 0 & 0 & \phi_{2,2,1,t} & \phi_{2,2,2,t} & 1 - \phi_{2,2,1,t} - \phi_{2,2,2,t} \\ 0 & 0 & 0 & 0 & 1 \end{bmatrix} \tag{13}$$

> *Phit0* := *Phit*:

> *B0* := ⟨⟨0|0|0|0|1⟩, ⟨1|0|1|0|0⟩, ⟨0|1|0|1|0⟩⟩;

$$B0 := \begin{bmatrix} 0 & 0 & 0 & 0 & 1 \\ 1 & 0 & 1 & 0 & 0 \\ 0 & 1 & 0 & 1 & 0 \end{bmatrix} \tag{14}$$

> *B* := ⟨⟨1 − *p*[1]|1 − *p*[2]|1 − *p*[1]|1 − *p*[2]|1⟩, ⟨*p*[1]|0|*p*[1]|0|0⟩, ⟨0|*p*[2]|0|*p*[2]|0⟩⟩ ;

$$B := \begin{bmatrix} 1 - p_1 & 1 - p_2 & 1 - p_1 & 1 - p_2 & 1 \\ p_1 & 0 & p_1 & 0 & 0 \\ 0 & p_2 & 0 & p_2 & 0 \end{bmatrix} \tag{15}$$

>

> #Then specify how many year of data and the number of sites, in this case there are T=5 years of data and N=2 sites.

```
> T := 5 : N := 2 :
```

> #Then input the unique histories for your data set. (The Mark format for this data set is given in simplesimexample.inp. The easiest way to do this from an .inp file is to open that file in excel and specify that there is a new column after every 0, 1 or 2, then copy and paste the data from excel, there is no need to input the [ ] brackets.)

$$H := \begin{bmatrix} 0 & 0 & 0 & 0 & 1 \\ 0 & 0 & 0 & 0 & 2 \\ 0 & 0 & 0 & 1 & 0 \\ 0 & 0 & 0 & 1 & 1 \\ 0 & 0 & 0 & 1 & 2 \\ 0 & 0 & 0 & 2 & 0 \\ 0 & 0 & 0 & 2 & 1 \\ 0 & 0 & 0 & 2 & 2 \\ 0 & 0 & 1 & 0 & 0 \\ 0 & 0 & 1 & 0 & 1 \\ 0 & 0 & 1 & 0 & 2 \\ 0 & 0 & 1 & 1 & 0 \\ 0 & 0 & 1 & 1 & 2 \\ 0 & 0 & 1 & 2 & 0 \\ 0 & 0 & 1 & 2 & 1 \\ 0 & 0 & 1 & 2 & 2 \\ 0 & 0 & 2 & 1 & 1 \\ 0 & 0 & 2 & 2 & 0 \\ 0 & 1 & 0 & 0 & 0 \\ 0 & 1 & 0 & 0 & 2 \\ 0 & 1 & 0 & 1 & 0 \\ 0 & 1 & 1 & 0 & 0 \\ 0 & 1 & 1 & 0 & 1 \\ 0 & 1 & 1 & 1 & 0 \\ 0 & 1 & 1 & 1 & 2 \\ 0 & 1 & 2 & 1 & 0 \\ 0 & 2 & 0 & 0 & 0 \\ 0 & 2 & 0 & 0 & 1 \\ 0 & 2 & 0 & 0 & 2 \\ 0 & 2 & 0 & 1 & 0 \\ 0 & 2 & 0 & 1 & 1 \\ 0 & 2 & 0 & 2 & 2 \\ 0 & 2 & 1 & 0 & 1 \\ 0 & 2 & 1 & 1 & 0 \end{bmatrix} :$$

```
> #We find the probabiltiies of each history using the code:
> kappa := Findkappa(H, Pit, Phi0, Phit, B0, B, T) :
> #Then we can find which parameters are present in the model using the following code:
> pars := ⟨seq(indets(kappa)[i], i = 1 ..nops(indets(kappa)))⟩ :
> #The derivative matrix is found using the following code:
> D1 := Dmat(kappa, pars) :
> #We find the rank and deficency using the hybrid method using the following code (the first
      number is the rank the second is the deficency):
> Hybrid(D1, pars);
```

$$\begin{bmatrix} 41 & 7 \end{bmatrix} \tag{16}$$

```
> #The deficency of this model is N^3 − N^2 = 4, but the deficency with this data set has increased
      to 7 (model is parameter redundant)
>
```
